# *MED27*, *SLC6A7,* and *MPPE1* Variants in a Complex Neurodevelopmental Disorder with Severe Dystonia

**DOI:** 10.1002/mds.29147

**Published:** 2022-07-25

**Authors:** Kimberley M. Reid, Robert Spaull, Smrithi Salian, Katy Barwick, Esther Meyer, Juan Zhen, Hiromi Hirata, Diba Sheipouri, Hind Benkerroum, Kathleen M. Gorman, Apostolos Papandreou, Michael A. Simpson, Yoshinobu Hirano, Irene Farabella, Maya Topf, Detelina Grozeva, Keren Carss, Martin Smith, Hardev Pall, Peter Lunt, Susanna De Gressi, Erik‐Jan Kamsteeg, Tobias B. Haack, Lucinda Carr, Rita Guerreiro, Jose Bras, Eamonn R. Maher, Richard H. Scott, Robert J. Vandenberg, F. Lucy Raymond, Wui K. Chong, Sniya Sudhakar, Kshitij Mankad, Maarten E. Reith, Philippe M. Campeau, Robert J. Harvey, Manju A. Kurian

**Affiliations:** ^1^ Molecular Neurosciences, Developmental Neurosciences, Zayed Centre for Research into Rare Disease in Children UCL Great Ormond Street Institute of Child Health London United Kingdom; ^2^ Department of Neurology Great Ormond Street Hospital London United Kingdom; ^3^ Department of Pediatrics, CHU Sainte‐Justine Research Center University of Montreal Montreal Quebec Canada; ^4^ Department of Psychiatry New York University School of Medicine New York New York USA; ^5^ Cell Therapy and Cell Engineering Facility Memorial Sloan Kettering Cancer Center New York New York USA; ^6^ Department of Chemistry and Biological Science College of Science and Engineering, Aoyama Gakuin University Sagamihara Japan; ^7^ School of Medical Sciences, University of Sydney Sydney New South Wales Australia; ^8^ Department of Neurology and Clinical Neurophysiology Children's Health Ireland at Temple Street Dublin Ireland; ^9^ School of Medicine and Medical Sciences University College Dublin Dublin Ireland; ^10^ Division of Genetics and Molecular Medicine King's College London School of Medicine London United Kingdom; ^11^ Leibniz Institute for Virology (HPI) and Universitätsklinikum Hamburg Eppendorf (UKE) Centre for Structural Systems Biology (CSSB) Hamburg Germany; ^12^ Institute of Structural and Molecular Biology, Crystallography/Department of Biological Sciences Birkbeck College, University of London London United Kingdom; ^13^ CNAG‐CRG, Centre for Genomic Regulation (CRG) The Barcelona Institute of Science and Technology (BIST) Barcelona Spain; ^14^ Department of Medical Genetics Cambridge Institute for Medical Research, University of Cambridge Cambridge United Kingdom; ^15^ Centre for Trials Research, Neuadd Meirionnydd Cardiff University Cardiff United Kingdom; ^16^ Wellcome Trust Sanger Institute Cambridge United Kingdom; ^17^ Department of Neurology John Radcliffe Hospital Oxford United Kingdom; ^18^ Department of Neurology Queen Elizabeth Hospital Birmingham United Kingdom; ^19^ Clinical Genetic Service Gloucester Royal Hospital Gloucester United Kingdom; ^20^ Department of Paediatrics Cheltenham General Hospital Gloucestershire United Kingdom; ^21^ Department of Human Genetics Radboud University Medical Center Nijmegen Netherlands; ^22^ Institute of Medical Genetics and Applied Genomics University of Tuebingen Tuebingen Germany; ^23^ Department of Neurodegenerative Science Van Andel Institute Grand Rapids Michigan USA; ^24^ Department of Medical Genetics University of Cambridge Cambridge United Kingdom; ^25^ Department of Clinical Genetics Great Ormond Street Hospital London United Kingdom; ^26^ Department of Radiology Great Ormond Street Hospital London United Kingdom; ^27^ Developmental Neurosciences Department UCL Great Ormond Street Institute of Child Health London United Kingdom; ^28^ School of Health and Behavioural Sciences University of the Sunshine Coast Sippy Downs Queensland Australia; ^29^ Sunshine Coast Health Institute Birtinya Queensland Australia

**Keywords:** *MED27*, *SLC6A7*, *MPPE1*, status dystonicus, dystonia

## Abstract

**Background:**

Despite advances in next generation sequencing technologies, the identification of variants of uncertain significance (VUS) can often hinder definitive diagnosis in patients with complex neurodevelopmental disorders.

**Objective:**

The objective of this study was to identify and characterize the underlying cause of disease in a family with two children with severe developmental delay associated with generalized dystonia and episodic status dystonicus, chorea, epilepsy, and cataracts.

**Methods:**

Candidate genes identified by autozygosity mapping and whole‐exome sequencing were characterized using cellular and vertebrate model systems.

**Results:**

Homozygous variants were found in three candidate genes: *MED27*, *SLC6A7*, and *MPPE1*. Although the patients had features of *MED27*‐related disorder, the *SLC6A7* and *MPPE1* variants were functionally investigated. *SLC6A7* variant *in vitro* overexpression caused decreased proline transport as a result of reduced cell‐surface expression, and zebrafish knockdown of *slc6a7* exhibited developmental delay and fragile motor neuron morphology that could not be rescued by L‐proline transporter–G396S RNA. Lastly, patient fibroblasts displayed reduced cell‐surface expression of glycophosphatidylinositol‐anchored proteins linked to *MPPE1* dysfunction.

**Conclusions:**

We report a family harboring a homozygous *MED27* variant with additional loss‐of‐function *SLC6A7* and *MPPE1* gene variants, which potentially contribute to a blended phenotype caused by multilocus pathogenic variants. © 2022 The Authors. *Movement Disorders* published by Wiley Periodicals LLC on behalf of International Parkinson and Movement Disorder Society

Next‐generation sequencing technologies have significantly improved the identification of new genetic diseases. Among others, the National Institute for Health and Care Research (NIHR) BioResource, Deciphering Developmental Disorders, and 100,000 genomes projects have pioneered seminal research, securing diagnoses for more than 6000 patients and driving the discovery of more than 200 new neurodevelopmental disorders.^1^, ^2^, ^3^ It is estimated that a genetic diagnosis can be identified for up to 60% of patients with development disorders.^4^

Despite these genetic advances, a significant proportion of children with neurodevelopmental disorders remain undiagnosed, and diagnosis is achieved in only 20% of patients with dystonia^5^; this reflects the current limitations of exome and genome sequencing and the challenges in determining the pathogenicity of variants of undetermined significance.^5^

We sought to investigate a consanguineous family with a genetically unresolved neurodevelopmental disorder characterized by severe global developmental delay, progressive dystonia and epilepsy. In this family, we identified homozygous variants in three genes: *MED27*, encoding the Mediator Complex Subunit 27 protein; *SLC6A7*, encoding the brain‐specific L‐proline transporter (PROT); and *MPPE1*, encoding Metalloproteinase 1 (PGAP5), an intracellular transporter of glycophosphatidylinositol (GPI)‐anchored proteins (GPI‐APs).

## Subjects and Methods

See Supplementary Methods for details.

## Results

### Clinical Characterization Shows a Complex Neurodevelopmental Phenotype with a Severe Hyperkinetic Movement Disorder, Epilepsy, and Cataracts

**FIG 1:**
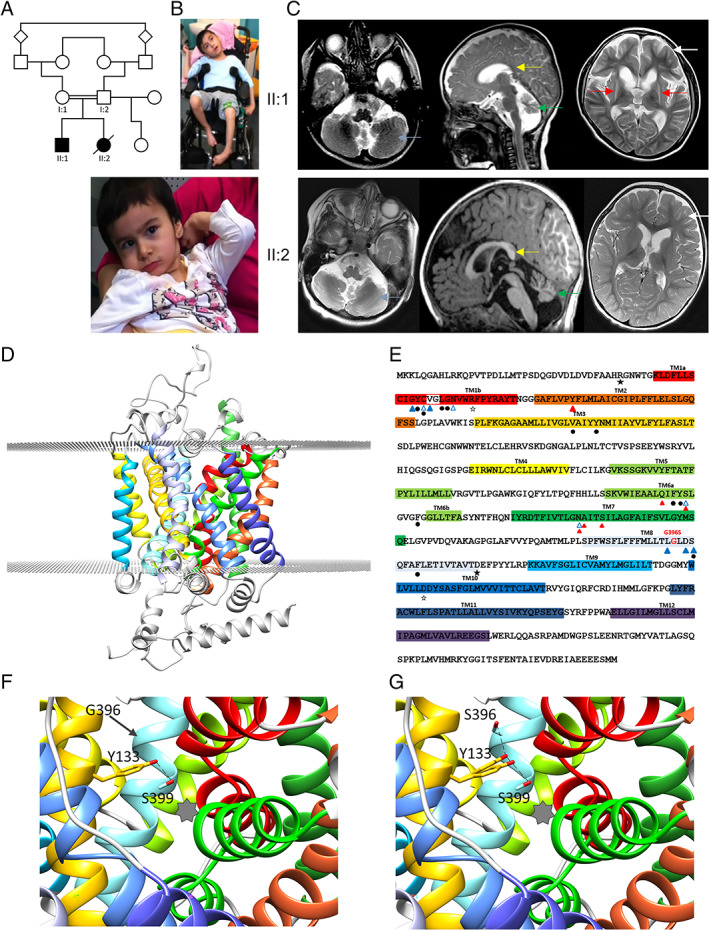
Clinical findings in sibship and the effect of the human L‐proline transporter (hPROT)‐G396S mutation on proline transporter structure and function. (**A**) Family pedigree, with affected individuals indicated by black shading. (**B**) Images of patient II:1 at 7 years old supported in a wheelchair with dystonic posturing of all limbs and patient II:2 at 4 years old sitting with support with dystonic upper limb posturing. Both have distinctive facial features with prominent eyebrows, slightly pointed noses, almond‐shaped eyes, and unusual‐shaped, low‐set ears. (**C**) Brain magnetic resonance imaging studies. Top row: patient II:1, axial (left) and sagittal T2‐weighted sequences (middle) at the age of 7 months, showing cerebellar hypoplasia (blue arrow) and small vermis (green arrows). The corpus callosum is vertically orientated posteriorly (yellow arrow). Repeat T2‐weighted axial sequences at the age of 10 years (right) show bilateral severe putaminal volume loss and T2 hyperintensity suggestive of gliosis (red arrows). Caudate volume loss is also seen without signal change. Enlarged frontal horns and subarachnoid spaces suggestive of bilateral frontal lobe atrophy are also seen (white arrow). Bottom row: patient II:2, age 2 years 4 months: T2 axial (left) and sagittal T1‐weighted sequences (middle) showing cerebellar hypoplasia (blue arrows) and small vermis (green arrow). The corpus callosum is vertically orientated posteriorly (yellow arrow). Relative frontal volume loss with white matter volume reduction is also appreciable on the axial T2 (right, white arrow). (**D**–**G**) Modeling of the hPROT transporter. (**D**) PROT consists of 12 transmembrane helices (highlighted in colors; transmembrane domain [TM] 1–TM12). (**E**) PROT amino acid sequence. p.Gly396Ser is located in TM8, adjacent to conserved residues located in the binding pocket. Blue triangles (outlined and filled) represent predicted residues involved in coordinating sodium ion Na1 and Na2 binding, respectively; red triangles represent residues predicted to be involved in chloride ion binding; black dots represent predicted residues important for proline binding; and black stars (outlined and filled) represent charged pairs at the extracellular and cytoplasmic entrances, respectively. (**F**) Structure modeling predicts that Gly396 (G396, TM8, cyan) is in close proximity to Tyr133 (Y133, TM3, orange), a highly conserved residue that is involved in substrate recognition. The star indicates the substrate binding pocket. (**G**) The introduction of serine with a hydroxyl group at position 396 (TM8, cyan) may alter substrate recognition activity of Y133 by introducing a different group to this region that could engage in H‐bonding with Y133, thereby interfering with its H‐bonding with S399 (two probable alternative rotamers for Y133 are shown). [Color figure can be viewed at wileyonlinelibrary.com]

Two siblings born to consanguineous parents (Fig. 1A) presented similarly with a complex neurodevelopmental disorder (see Supplementary Results). Both had infantile hypotonia with progressive generalized dystonic and choreiform movements that were refractory to medical treatment. Both had episodic status dystonicus requiring intensive care. Both children developed focal epilepsy by age 4 years, required lensectomy for cataracts in mid childhood, and had gastrointestinal dysmotility. Neither achieved the ability to sit unsupported or communicate. On clinical examination, both had distinctive facial features, microcephaly, significant axial hypotonia, generalized dystonia, torticollis, intermittent opisthotonus and distal choreoathetosis (Fig. 1B, Videos S1 and S2).

Early brain magnetic resonance imaging was similar for both, showing minimal underdevelopment of the white matter and cerebellar hypoplasia with a small vermis (Fig. 1C). The older sibling (II:1) had repeat neuroimaging at 10 years that showed symmetrical bilateral atrophy of the striatum associated with signal changes suggestive of gliosis, as well as frontal‐predominant cerebral volume loss (Fig. 1C).

### Molecular Genetic Analysis Identifies Candidate Gene Variants in *MED27* , *SLC6A7* , and *MPPE1*

Homozygous variants were identified in three candidate genes (*MED27*, *SLC6A7*, and *MPPE1*) from exome sequencing of patient II:2 combined with single‐nucleotide polymorphism (SNP) genotyping (see Supplementary Results). The *MED27* variant NM_004269.3; c.839C>T (p.Pro280Leu) affects a highly conserved amino acid (Fig. S1A) and has previously been reported in patients with similar disease phemonology.^6^ The *SLC6A7* variant NM_014228.4: c.1186G>A (p.Gly396Ser) affects a highly conserved amino acid, both throughout species and among other SLC6 transporters (Fig. S1B). The variant is known to genomic databases (Table S4) but has not been previously reported in homozygous state. The *MPPE1* variant NM_023075.5: c.985A>T (p.Arg329*) is predicted to eliminate 67 amino acid residues from the C‐terminus of PGAP5 (Fig. S1C) and occurs before the integral transmembrane domain (TM) that anchors the protein into the endoplasmic reticulum (ER) and Golgi membrane, and the ER retention signal (KxKxx) which is required for correct localization^7^ (Fig. S2).

### Homology Modeling of hPROT‐G396S Predicts Altered Substrate Recognition and Protein Destabilization

PROT, encoded by *SLC6A7*, is part of the SLC6 family of transporters,^8^ which are integral to the transport of neurotransmitters, amino acids, and monoamines against their concentration gradient, with symport of Na^+^ and Cl^−^. The AlphaFold‐based model^9^ (Fig. 1D) showed that the highly conserved residue hPROT‐G396 is located in TM8, flanked by several residues important for both Na^+^ and substrate binding (Fig. 1E), such as Y133 in TM3 (Fig. 1F), a highly conserved residue in the SLC6 family. This tyrosine residue is engaged in an H‐bond with Ser399, another highly conserved residue that interacts with the substrate. The p.Gly396Ser substitution in PROT introduces a hydroxyl group to this region that could engage in H‐bonding with Y133, altering the electrostatics of the pocket where the proline substrate binds, thereby affecting its binding affinity (Fig. 1G).

### hPROT‐G396S Is Associated with Decreased Cell‐Surface Protein Expression, Reduced Proline Affinity, and Impaired Proline Transport

**FIG 2:**
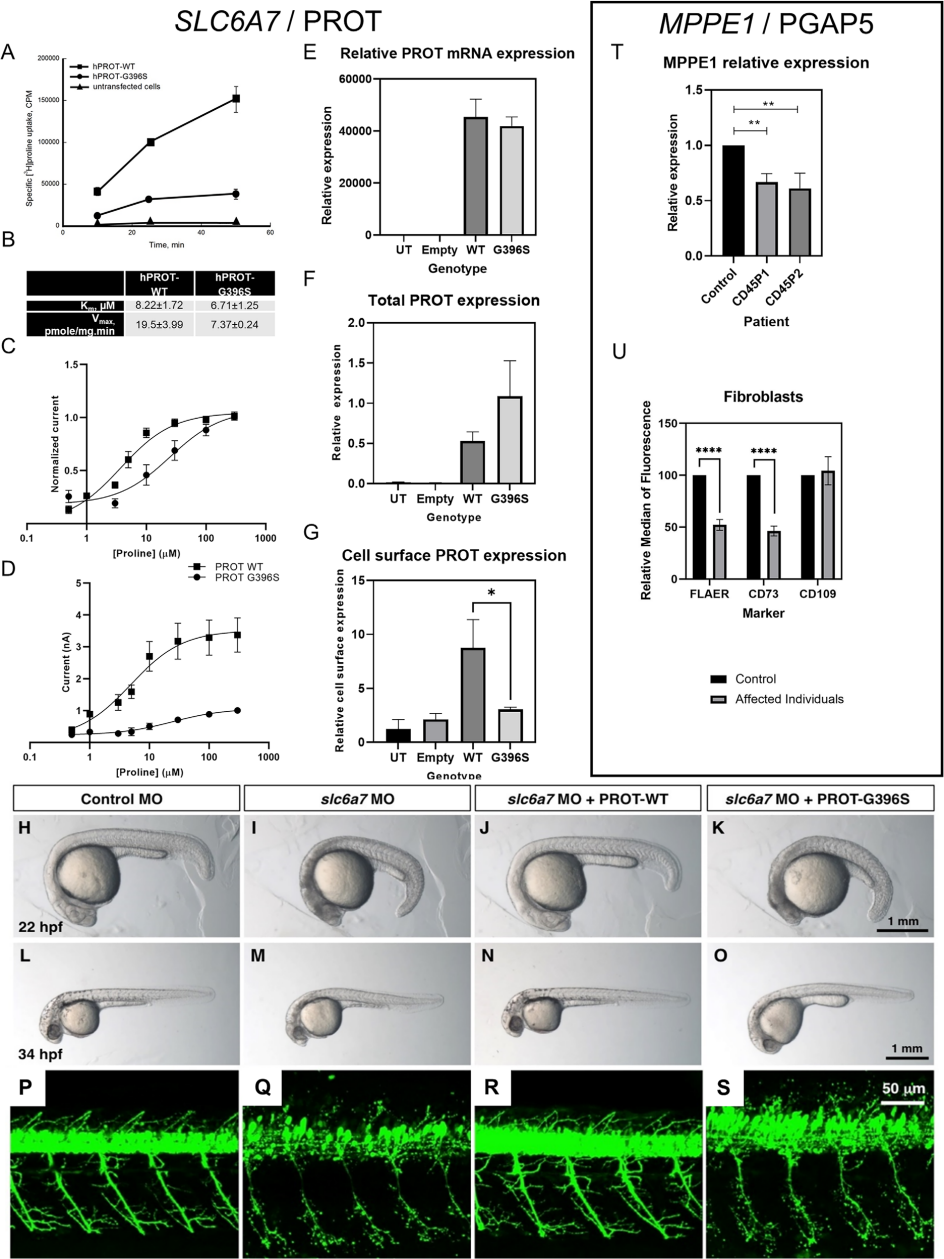
Functional investigations into *SLC6A7* and *MPPE1* gene variants. (**A**, **B**) Time‐course experiments of [^3^H]L‐proline uptake show decreased L‐proline accumulation in LLC‐PK cells expressing L‐proline transporter (hPROT)‐G396S in comparison with hPROT‐WT (wild type) (**A**) with decreased maximal uptake velocity (*V*_max_) but no differences in *K*_m_, n = 4 (**B**). (**C**, **D**) Proline dose responses were measured in *Xenopus* oocytes expressing WT (n = 5) or G396S (n = 6) hPROT. (**C**) hPROT‐G396S had reduced apparent affinity for proline (EC_50_ = 22.05 ± 9.21) compared with WT (EC_50_ = 4.86 ± 0.49). (**D**) hPROT‐G396S also had reduced maximal currents (*I*_max_ = 1.01 nA ± 0.03) compared with WT (*I*_max_ = 3.37 ± 0.54 nA). All values reported are mean ± standard error of the mean. (**E**) PROT mRNA expression in HEK293T cells. No significant differences between WT and mutant mRNA expression were evident. (**F**, **G**) Biotinylation and immunoblotting analysis showed no significant difference in total protein expression but significantly less expression of hPROT‐G396S at the cell surface compared with hPROT‐WT in transiently transfected HEK‐293T cells (*P < 0.05, Student *t* test). (**H**–**O**) Knockdown of *slc6a7* in zebrafish caused a delay in development. Control morpholino oligonucleotide (MO)‐injected larvae were equivalent to 21.9 ± 0.1 hours post fertilisation (hpf) stage (**H**; n = 40), while *slc6a7* morphant development correlated to the 18.6 ± 0.2 hpf stage (**I**; n = 47). This developmental delay was reversed in zebrafish larvae coinjected with *slc6a7* MO and human PROT‐WT RNA to 21.8 ± 0.1 hpf stage at 22 hpf (**J**; n = 40). The development of zebrafish coinjected with *slc6a7* MO and human PROT‐G396S RNA was decelerated to 18.6 ± 0.3 hpf stage at 22 hpf (**K**; n = 45). Similar observations were made at 34 hpf, where the development of control MO‐injected larvae is equivalent to 33.6 ± 0.3 hpf stage (**L**; n = 51), while *slc6a7* morphant development correlated to 29.0 ± 0.4 hpf stage (**M**; n = 70). The development of zebrafish larvae coinjected with *slc6a7* MO and human PROT‐WT RNA was equivalent to 33.2 ± 0.3 hpf stage at 34 hpf (**N**; n = 51), while zebrafish coinjected with *slc6a7* MO and human PROT‐G396S RNA developed to 29.3 ± 0.4 hpf stage (**O**; n = 70). (**P**–**S**) MO‐mediated knockdown of *slc6a7* in transgenic zebrafish expressing YFP in motor neurons resulted in fragile motor neuron morphology (**Q**) compared with controls (**P**). This motor neuron phenotype was reversed by coinjection of hPROT‐WT RNA (**R**), but not by introduction of hPROT‐G396S RNA (**S**). (**T**) qPCR of *MPPE1* mRNA in patient and healthy control fibroblasts (n = 3 using one healthy age‐ and sex‐matched control and two patient lines; **P < 0.01, Student *t* test). (**U**) Cell‐surface expression of GPI‐APs shows that FLAER and CD73 were significantly reduced in patient fibroblasts (n = 6, cells from both patients) compared with control samples (n = 3); ****P < 0.0001 (unpaired parametric *t* test). No significant differences were seen for CD109. [Color figure can be viewed at wileyonlinelibrary.com]

To determine the impact of p.Gly396Ser on PROT function, we measured L‐proline uptake in LLC‐PK_1_ cells transiently expressing either hPROT‐WT or hPROT‐G396S. The time course of [^3^H]L‐proline uptake indicated near‐linear uptake for up to 25 minutes (Fig. 2A). At 10 minutes, hPROT‐G396S displayed greatly reduced transport activity, at a level ~30% of hPROT‐WT (Fig. 2A,B) with reduction in maximal uptake velocity (*V*_max_) (Fig. 2B). These findings were confirmed in *Xenopus* oocytes; hPROT‐G396S showed a reduced apparent affinity for proline (EC_50_ = 22.05 ± 9.21) and reduced maximal currents (*I*_max_ = 1.01 nA ± 0.03) compared with wild‐type PROT (EC_50_ = 4.86 ± 0.49, *I*_max_ = 3.37 ± 0.54 nA) (Fig. 2C,D). In HEK293T cells, no significant difference was seen in mRNA or total protein levels of hPROT‐WT and hPROT‐G396S (Fig. 2E,F, Fig. S3). However, biotinylation studies demonstrated that hPROT‐G396S showed significantly reduced cell‐surface expression (Fig. 2G, Fig. S3).

### Morpholino Knockdown of *slc6a7* in Zebrafish Leads to Delayed Development and Fragile Motor Neuron Morphology

To determine effects *in vivo*, we used antisense morpholino oligonucleotides (MOs) against *slc6a7* in a zebrafish model. When compared with control MO‐injected larvae (Fig. 2H,L), *slc6a7* morphants showed delayed motor development (Fig. 2I,M). Coinjection of *slc6a7* MO with human PROT‐WT RNA restored normal development (Fig. 2J,N), whereas coinjection with PROT‐G396S did not show recovery (Fig. 2K,O). Developmental stage was delayed in *slc6a7* morphants and *slc6a7* morphants injected with PROT‐G396S RNA, but not in *slc6a7* morphants injected with PROT‐WT RNA (Fig. S4). Furthermore, motor neurons of the MO‐injected zebrafish larvae displayed fragile morphology, with a reduced cell body number (Fig. 2P–S). Coinjection of *slc6a7* MO with PROT‐WT RNA led to recovery of this motor neuron phenotype (Fig. 2R), whereas no rescue was observed in *slc6a7* morphants coinjected with PROT‐G396S RNA (Fig. 2S).

### Patient Fibroblasts Display Altered Cell‐Surface Expression of GPI‐APs

PGAP5, encoded by *MPPE1*, translocates from the Golgi to the ER through an ER retrieval signal at its C‐terminus (Fig. S5). A side‐chain ethanolamine‐phosphate from the second mannose molecule of the GPI is removed, allowing GPI‐AP to exit from the ER (Fig. S5). Without this step, GPI‐APs are retained in the ER.^10^ MPPE1 expression was significantly reduced in patient fibroblasts (*P* < 0.01, Student *t* test) (Fig. 2T). Flow cytometry was undertaken to assess cell‐surface expression of GPI‐APs. Flourescently labelled aerolysin (FLAER) serves as a marker of total GPI‐APs because it binds directly to the GPI anchor, while CD73 and CD109 are specific GPI‐APs. Cell‐surface levels of FLAER and CD73 were significantly reduced in both patients (Fig. 2U, Fig. S6). No decrease in the level of CD109 was observed.

## Discussion

We report two siblings born to consanguineous parents presenting with a severe and progressive neurodevelopmental disorder. Molecular genetic analysis windicated three plausible gene candidates with homozygous variants in *MED27*, *SLC6A7*, and *MPPE1*. *MED27* encodes subunit 27 of the Mediator of RNA polymerase II Transcription (Mediator) complex, which mediates RNA polymerase II transcription.^11^ Biallelic *MED27* variants have recently been reported in 11 families with a complex neurodevelopmental disorder that partially overlaps with our cases^6^ (Table S8). *MED27* is therefore the most convincing candidate gene. However, the presence of chorea, severity of dystonia with recurrent status dystonicus, and striatal atrophy distinguishes our patients from reported *MED27* cases (Table S8). It is thus plausible that the additional loss‐of‐function variants in *SLC6A7* and *MPPE1* could contribute to disease.

*SLC6A7* encodes a the central nervous system protein, PROT (Table S9).^12^ PROT mediates the high‐affinity uptake of L‐proline into glutamatergic neurons, maintaining an intracellular pool of L‐proline for glutamate production.^13^, ^14^, ^15^ The p.Gly396Ser substitution perturbs normal PROT function. Notably, diseases associated with abnormal proline homeostasis and hyperprolinemia commonly present with neurological features.^16^, ^17^ Defective PROT and proline dyshomeostasis is also associated with neurodevelopmental defects in animal models, as evident in our zebrafish MO knockdowns. Although morpholinos can be associated with nonspecific phenotypes, rescue with wild‐type PROT, but not G396S PROT, RNA provides evidence of specificity. The PROT knockout mouse model also shows reduced locomotor activity, decreased approach motivation, and impaired memory extinction.^18^

*MPPE1* encodes PGAP5, a widely expressed metalloproteinase^19^ integral to the GPI biosynthesis and protein‐anchoring pathway, a highly conserved eukaryotic posttranslational modification.^10^ The addition and modification of a GPI anchor is essential for the trafficking of certain proteins from the ER to the Golgi and cell surface.^20^, ^21^ The *MPPE1* variant p.Arg329* likely leads to translation of a truncated protein lacking the KxKxx ER retrieval signal, which is crucial for correct PGAP5 localization.^7^ We postulate that this would reduce cell‐surface expression of GPI‐APs (FLAER and CD73). Notably, there was no significant reduction in CD109. Such variation in affected GPI‐APs is observed in other GPI biosynthesis disorders, where the specific gene and mutation influence the pattern of GPI‐AP disturbance.^22^, ^23^, ^24^, ^25^

It is highly likely that these *SLC6A7* and *MPPE1* variants contribute to the observed clinical phenotype. Both SLC6 transportopathies and inherited disorders of GPI deficiency are associated with a broad range of neurological diseases.^26^, ^27^ Early neuroimaging abnormalities in our patients are similar to those described in *MED27*‐related disease^6^ and GPI deficiency disorders with cerebellar defects and white matter changes.^27^ To date, the later imaging findings in the older sibling have not been reported in *MED27* disease. Striatal atrophy and gliosis may be sequelae of status dystonicus with multiorgan failure, but the influence of mutant *MPPE1* and *SLC6A7* cannot be excluded. Indeed, this pattern of damage closely matches the expression pattern of *SLC6A7*.^28^

Multilocus pathogenic variants have been previously described in consanguineous families^29^ and are likely to explain a proportion of unresolved Mendelian disorders.^30^ Phenotypic features atypical for *MED27*, such as the severe movement disorder and striatal atrophy, may be explained by these additional variants resulting in a blended phenotype.^31^, ^32^ Future identification of patients with monogenic variants in *SLC6A7* or *MPPE1* will undoubtedly facilitate better understanding of the precise gene‐specific clinical phenotypes.

In conclusion, we have identified variants in *MED27*, *SLC6A7*, and *MPPE1* in a family with a complex and severe neurodevelopmental condition associated with a life‐threatening movement disorder. Pathogenic variants in *SLC6A7* and *MPPE1* have not previously been reported in human disease. Our work not only suggests that variants in these genes may be relevant in human disease but also indicate a putative role for PROT and PGAP5 in normal neurodevelopment. Further experimental approaches with animal models or patient induced pluripotent stem cell (iPSC)‐derived neuronal systems harboring all three genetic variants will allow the polygenic influence of these three genes to be investigated.

## Author Roles

(A) Design, (B) execution, (C) analysis, (D) writing, and (E) editing of final version of the manuscript.

K.M.R.: A, B, C, D, and E.

R.S.: B, C, D, and E.

S. Salian: B and C.

K.B.: B, C, D, and E.

E.M.: A, B, and C.

J.Z.: B and C.

H.H.: A, B, and C.

D.S.: B and C.

H.B.: B and C.

K.M.G.: B.

A.P.: B.

M.A.S.: C.

Y.H.: B.

I.F.: B.

M.T.: A, B, and C.

D.G.: C.

K.C.: C.

M.S.: B.

H.P.: B.

P.L.: B.

S.D.G.: B.

E.‐J.K.: C.

T.B.H.: C.

L.C.: B.

R.G.: C.

J.B.: C.

E.R.M.: A.

R.H.S.: B.

F.L.R.: C.

W.K.C.: C.

R.J.V.: A, B, and D.

S. Sudhakar: C.

K.M.: C.

M.E.R.: A, B, and C.

P.M.C.: A, B, C, and E.

R.J.H.: A, B, C, D, and E.

M.A.K.: A, B, C, D, and E.

## Financial Disclosures

K.M.R.: Salary supported by grant from the NIHR, with research supported by grants from NIHR and Rosetrees trust. R.S. and S. Salian: Salary supported by grants from the NIHR, Great Ormond Hospital Children's Charity, and LifeArc. K.B., E.M., J.Z., and H.H.: Salary supported by grant from the NIHR, with research supported by grants from NIHR, Sir Jules Thorne, and Rosetrees trust. D.S. and H.B.: Supported by a University of Sydney Research Training Program Scholarship. K.G.: Supported by the Temple Street Foundation. A.P., M.A.S., Y.H., I.F., and M.T.: Research supported by a joint AMR/BPNA Clinical Research Training Fellowship (GN 2465) and an NIHR (GOSH BRC) Catalyst fellowship, as well as grants from Actelion, Rosetrees Trust, and NBIA Disorders Association. D.G., K.C., M.S., H.P., P.L., S.D.G., and E.‐J.K.: Salary supported by the National Institute for Health Research and UK Research and Innovation. T.B.H., L.C., R.G., and J.B.: Supported by funding from the German Research Foundation. E.R.M., R.H.S., F.L.R., and W.K.C.: Funding from the NIHR Cambridge Biomedical Research Centre. The University of Cambridge has received salary support (E.R.M.) from the NHS in the East of England through the Clinical Academic Reserve. R.J.V., S. Sudhakar, K.M., and M.E.R.: NHMRC grant APP144429 and National Institutes of Health grant RO1 4219209. P.M.C.: Received research funding from the CIHR (Canadian Institutes of Health Research) for research unrelated to the present study. R.J.H.: MRC grant M013502 and NHMRC grant APP1156673. M.A.K.: Funding supported from NIHR Research Professorship, Sir Jules Thorn Award for Biomedical Research, and the Rosetrees Trust. The authors declare no potential conflicts of interest.

## Supporting information


**Video S1** Patient II:1. Age 4 years: Axial hypotonia and dystonic posturing of the left hand. Age 7 years: Generalized dystonia, with upper limb postures (hand fisting) and lower limb posturing (striatal toe, foot clawing). Age 9 years: An overall paucity of limb movement and facial expression. Opisthotonic posturing is seen, as well as dystonic postures of the upper and lower limbs.

**Video S2** Patient II:2. Age 2 years: Axial hypotonia with dystonic postures of the upper limbs. Occasional choreoathetoid movements of the limbs are noted with mouthing and orolingual dyskinesia. Age 4 years: Axial hypotonia with dystonic posturing of all four limbs and intermittent striatal toe. Tongue protrusion with orolingual dyskinesia.

**Appendix S1** Supporting Information

**FIG. S1** MED27, SCL6A7 and MPPE1 variant conservation.

**FIG. S2** Gene and protein schematic of MPPE1/PGAP5.

**FIG. S3** Biotinylation assays and Western blots of p.Gly396Ser variant overexpressed in HEK293T cells.

**FIG. S4** Developmental stage of sclc6a7 morphants.

**FIG. S5** Schematic of PGAP5 function.

**FIG. S6** Flow cytometry plots of cell‐surface GPI‐AP expression (CD73, CD109 and FLAER) in patient fibroblasts.

## Data Availability

The data that supports the findings of this study are available in the supplementary material of this article
