# Supplementary material for: MED27, SLC6A7, and MPPE1 Variants in a Complex Neurodevelopmental Disorder with Severe Dystonia
Source: Mov Disord. 2022 Jul 25;37(10):2139–46. doi: 10.1002/mds.29147 (PMC9796674; doi:10.1002/mds.29147)
Supplement: Supplementary file 3 — Appendix S1 Supporting Information [file MDS-37-2139-s007.docx]

***MED27, SLC6A7* and *MPPE1* variants in a complex neurodevelopmental disorder with severe dystonia**

**Authors:**

Kimberley M. Reid PhD^1^, Robert Spaull MRCPCH^1,2^, Smrithi Salian PhD^3^, Katy Barwick PhD^1^, Esther Meyer PhD^1^, Juan Zhen PhD^4,5^, Hiromi Hirata PhD^6^, Diba Sheipouri PhD^7^, Hind Benkerroum MSc^3^, Kathleen Gorman MD^8,9^, Apostolos Papandreou PhD^1,2^, Michael A. Simpson PhD^10^, Yoshinobu Hirano PhD^6^, Irene Farabella PhD^11,12,13^, Maya Topf DPhil^11,12^, Detelina Grozeva PhD^14,15^, Keren Carss PhD^16^, Martin Smith PhD^17^, Hardev Pall MD^18^, Peter Lunt FRCP^19^, Susanna De Gressi MRCPCH^20^, Erik-Jan Kamsteeg PhD^21^, Tobias B. Haack PhD^22^, Lucinda Carr MD^2^, Rita Guerreiro PhD^23^, Jose Bras PhD^23^, Eamonn R. Maher FMedSci^24^, Richard H. Scott PhD^25^, Robert J. Vandenberg^7^, F. Lucy Raymond PhD^15^, Wui K. Chong MD^26,27^, Sniya Sudhakar^26,27^, Kshitij Mankad FRCR^26,27^, Maarten E. Reith PhD^4^, Philippe M. Campeau MD^3^, Robert J. Harvey PhD^28,29^, Manju A. Kurian PhD^1,2,*^

1. Supplementary Methods Page 2
2. Supplementary Results Page 6
   1. Case vignette
   2. Candidate variant identification
3. Supplementary Tables Page 8
4. Supplementary Figure Legends Page 20
5. Supplementary References Page 21

**A. Supplementary Methods**

**Molecular genetic analysis**

**Subjects**

A consanguineous family with two affected children was recruited for molecular genetic investigation. Written informed consent was obtained for all study participants. The study was approved by the National Research Ethics Service in the United Kingdom (National Research Ethics Service Committee: London-Bloomsbury, REC reference: 13/LO/0168), and performed in accordance with the Declaration of Helsinki. Written informed consent was obtained for publication of patient videos. Genomic DNA and skin fibroblasts from participating individuals was extracted using standard techniques.

**Molecular genetics**

SNP genotyping was performed in both parents and the two affected children. Whole Exome Sequencing (WES) was performed in Patient II:2. Sanger Sequencing confirmed genetic variants and familial segregation. Six genomic databases (approximately 6500 exomes) were screened for variants in *SLC6A7* and *MPPE1* (**Table S1**).

**SNP genotyping**

Based on familial consanguinity, an autozygosity mapping strategy was applied. SNP genotyping was performed in both parents and the two affected children using Illumina cytoSNP-12 (Illumina Inc., San Diego, CA). Genotype data were processed with Genomestudio (Illumina Inc.) and subsequently analyzed using both BEDTools^1^ and manually in Microsoft Excel.

**Whole Exome Sequencing (WES)**

WES was analysed using the SureSelect All Exon 50Mb Target Enrichment System/SureSelect Human All Exon kit (v2; Agilent Technologies), was carried out in affected individual II:2 on the Illumina GaIIx with 76bp paired-end reads. The sequences were aligned to the reference human genome (hg19 build) with Novoalign (Novocraft Technologies Sdn Bhd). After omitting duplicate reads (caused by PCR clonality or optical duplicates) and reads mapping to multiple sites, the depth and breadth of sequence coverage was calculated using custom scripts and the BedTools package. Detected single nucleotide substitutions and small insertion deletions were quality filtered within the SamTools software package and in-house software tools excluding calls with a read coverage <4x and a Phred-scaled SNP quality of <20. Subsequently ANNOVAR was used to annotate variants with respect to genes and transcripts. Novelty of variants was determined by comparison to dbSNP132, 1000 Genomes SNP calls and a subset of 250 control exomes generated by the same method.

**Direct Sanger sequencing**

To confirm the variants identified on whole exome sequencing and to establish familial segregation, direct Sanger sequencing was undertaken. The genomic DNA sequence of the respective genes were taken from Ensembl (<http://www.ensembl.org/index.html>) and primer pairs for relevant variants were designed with Primer3Plus software^2^ (<https://www.bioinformatics.nl/cgi-bin/primer3plus/primer3plus.cgi>) (**Table S10**). PCR amplification was performed with BioMix™ Red (Bioline Ltd.). PCR products were purified with MicroCLEAN (Web Scientific) and directly sequenced by the Big Dye Terminator Cycle v1.1 Sequencing System (Applied Biosystems Inc.). Sequencing reactions were run on an ABI PRISM 3730 DNA Analyzer (Applied Biosystems Inc.) and analyzed using Chromas and Mutation Surveyot. (<http://www.technelysium.com.au/chromas.html>).

**Functional investigations**

**Site-directed mutagenesis and transfection**

The human proline transporter plasmid (cat# RC210838) was purchased from OriGene Technologies, InC. (Rockville, MD, USA). Site-directed mutagenesis was performed using the QuikChange site-directed mutagenesis kit as previously described^3^. Culturing and transient transfection of HEK-293T or LLC-PK_1_ cells with Lipofectamine 2000 (Thermo Fisher Scientific Inc., Waltham, MA, USA) were carried out as previously described^3^. Proline uptake assays, PROT surface expression and analysis of MPPE1 gene expression were performed.

**Proline uptake studies**

LLC-PK_1_ cells transiently expressing hPROT-WT or hPROT-G396S were used for functional [^3^H]L-proline uptake assays as previously described^4^. For saturation analysis, different concentrations of non-radioactive L-proline spanning its K_m_ (0.3-100 µM) and one fixed concentration of [^3^H]L-proline (~4 nM) were used. The K_m_ and V_max_ of L-proline uptake were calculated by non-linear regression fitting using the Radlig software (KELL program from Biosoft, Great Shelford, Cambridge, United Kingdom). Comparisons of hPROT functional activity between WT and the G396S mutant were performed with an unpaired parametric Student’s t-test. Statistical significance was considered where P<0.05.

**PROT surface protein expression by biotinylation**

Biotinylation assays on HEK-293T cells transiently expressing either hPROT-WT or hPROT-G396S were conducted as previously described^4^. hPROT was probed with polyclonal rabbit anti-FLAG antibody (Sigma-Aldrich, St. Louis, MO, USA) against the FLAG tag fused to C-terminus of hPROT, followed by HRP-conjugated goat anti-rabbit antibody. Polyclonal anti-β-actin antibody (Sigma-Aldrich, St. Louis, MO, USA) and anti Na^+^/K^+^ 𝛼 ATPase antibody (Abcam) were used as a loading control for the total cell lysate and membrane bound fractions, respectively. The transporter signal was visualized using SuperSignal West Dura Extended Duration chemiluminescence substrate solution (Thermo Scientific). The relative fraction of glycosylated and non-glycosylated hPROT was normalized by the total amount of the two isoforms, and comparisons performed using an unpaired parametric Student’s t-test. Statistical significance was considered where P<0.05.

**Analysis of MPPE1 gene expression**

RNA was extracted from cultured fibroblasts using the Rneasy Mini kit (Qiagen). First-strand cDNA synthesis was carried out with SuperScript III Reverse Transcriptase (Invitrogen) using 1μg of total RNA per reaction and oligo(dT) primers (Thermo Fisher Scientific). Changes in the relative expression of *MPPE1* was measured by qRT–PCR on a StepOnePlus Real-Time PCR System (Applied Biosystems). RT–PCR reactions comprised 1× MESA Blue qPCR MasterMix Plus for SYBR Assay (Eurogentec), 0.1 μl of ROX Reference Dye (Invitrogen), 9 μl of cDNA (of a 1:25 dilution) and 500 nM of each primer (**Table S11**)**.** RT–PCR conditions are available upon request. Relative quantification of gene expression was performed using the 2−ΔΔCt method^5^, with GAPDH (glyceraldehyde-3-phosphate dehydrogenase) as a reference gene.

**Molecular modelling**

The model of the proline transporter PROT (SLC6A7_HUMAN) was downloaded from the AlphaFold Structure Database^6,7^<https://alphafold.ebi.ac.uk/>). The non-synonymous substitution p.Gly396Ser was modelled with the swappa command in UCSF Chimera^8^ and the most probable rotamers were selected using the Dunbrack rotamer library^9^ and with Chimera’s default settings. The positioning of PROT in membranes was predicted using the PPM 3.0 Web Server^10^ with default settings.

**Animals**

Zebrafish experiments were carried out in compliance with the guidelines approved by the animal care and use committee in the National Institute of Genetics and Aoyama Gakuin University (Japan). Zebrafish (Danio rerio) of the transgenic Tg(hb9:Venus)nig2 (National BioResource Project Zebrafish, Japan) expressing modified YFP in motor neurons were maintained at 28.5°C with constant light-dark cycles and fed twice daily following established procedures^11^.

**GPI-Anchored protein investigations in patient fibroblasts**

To evaluate cell surface expression of GPI-anchored proteins (GPI-APs), flow cytometry analysis was performed on cultured fibroblasts derived from the affected siblings, three healthy age-matched controls (1 female, 2 males, mean age 10) and a positive control with PIGK deficiency^12^. Cells were probed for FLAER – an Alexa Fluor® 488 labeled variant of aerolysin, which binds specifically to mammalian GPI anchors (Cedarlane), FITC-conjugated mouse anti-human CD73 (BioLegend) and PE-conjugated mouse anti-human CD109 (BioLegend) as previously described^13^. The cells were analyzed by a BD FACSCanto II system (BD Bioscience) and Cytobank software was used to analyze the data. Comparisons were made using an unpaired parametric Students t-test.

**Xenopus oocyte studies**

Human wild-type and p.Gly396Ser PROT cDNAs were subcloned into the oocyte transcription vector pOTV and sequenced by the Australian Genome Research Facility (Sydney, Australia). Complementary RNA was expressed in *Xenopus laevis* oocytes as previously described^14^. Oocytes were voltage clamped at -60 mV, in frog Ringer's solution (96 mM NaCl, 2 mM KCl, 1 mM MgCl_2_, 1.8 mM CaCl_2_, 5 mM HEPES, pH 7.5) and whole-cell currents generated by the proline were recorded with a Geneclamp 500 amplifier (Axon Instruments, Foster City, California, USA) and a Powerlab 2/20 chart recorder (ADInstruments, Sydney, Australia).

**Knockdown and rescue experiments in zebrafish**

Antisense morpholino oligonucleotide (MO) was designed targeting the exon3-intron3 junction (5'- ATGCGTAAATGTACCTTTGAGGAGG -3') of the *SLC6A7* zebrafish ortholog, *slc6a7*, thereby inhibiting RNA splicing of mRNA, as well as a standard negative control MO (5'-CCTCTTACCTCAGTTACAATTTATA-3'). 5 ng of the MOs were delivered by microinjection into 1-2 cell stage embryos that were subsequently raised at 28.5°C. At 22 and 34 hours post fertilization (hpf), larvae morphology was observed and developing stage estimated as described^11^. The morphology of motor neurons was examined using a confocal microscope (TCS SP5 II, Leica). For rescue experiments, hPROT-WT and hPROT-G396S were cloned into the pCS2+ vector and transcribed into RNA using the mMESSAGE mMACHINE SP6 kit (Life Technologies). The RNAs were co-injected with *slc6a7* MO into 1-2-cell stage embryos and motor neurons analyzed at 36 hpf.

**B. Supplementary Results**

**Case vignette**

Two siblings born to consanguineous Turkish parents presented similarly with a complex neurodevelopmental disorder (**Figure 1A**). There was no relevant family history of neurological disorders. Uncomplicated maternal gestational diabetes was present in both pregnancies, and normal *in utero* movements were reported. Both were born at term by spontaneous vaginal delivery with no early concerns.

Severe truncal hypotonia and head lag were apparent from early infancy. By the age of 6 months, both siblings developed a progressive hyperkinetic movement disorder with generalized dystonia and chorea. Limited improvement in hyperkinesia was seen with Gabapentin, Trihexyphenidyl, Clobazam, Baclofen and Clonidine. Aged 9 years, the older sibling (II:1) presented with life-threatening status dystonicus triggered by an infection requiring intensive care and peritoneal dialysis for renal failure secondary to rhabdomyolysis. Similarly, aged 7 years, the younger sibling (II:2) developed a severe exacerbation of dystonia (painful dystonic posturing of limbs and trunk, sleep disturbance, difficulty in seating, and elevated creatine kinase levels) associated with a respiratory illness. Each have required admission to intensive care for status dystonicus since, and the severity of the generalized dystonia has resulted in seating difficulties and chronic sleep disturbance.

Both children developed epilepsy by age 4 years, manifesting initially as focal motor seizures with later generalized tonic-clonic seizures. EEG recordings revealed frequent spikes and/or sharp waves over the frontal/temporal regions bilaterally. Both responded well to Carbamazepine and/or Levetiracetam. Cataracts were detected on routine ophthalmological assessment in mid-childhood requiring bilateral lensectomy. Other systemic features include gastrointestinal dysmotility (drooling, feeding difficulties, gastro-esophageal reflux and vomiting necessitating gastrostomy insertion and Nissen’s fundoplication), and frequent chest infections.

On clinical examination, both had distinctive facial features (**Figure 1B**), microcephaly (head circumference below 0.4^th^ centile), significant axial hypotonia, generalized dystonia, torticollis, intermittent opisthotonus, variable limb tone with intermittent dystonic posturing of the limbs (**Figure 1B**) and distal choreoathetosis (**Video S1**, **S2**). Patient II:1 is alive (now 15 years), but Patient II:2 died age 12 years after a short respiratory illness.

**Candidate variant identification**

Single-nucleotide polymorphism (SNP) genotyping revealed five extended regions of common homozygosity shared by the two affected siblings (**Table S3**). Exome sequencing of patient II:2 revealed 23,166 variants that were prioritized as follows: (i) those within regions of shared homozygosity; (ii) non-synonymous, frameshift, splice site, and nonsense changes; (iii) absent or observed at a very low frequency as a heterozygous variant (<1%) in publicly available databases dbSNP, 1000 Genomes, ESP6500 and ExAc; (iv) affecting highly conserved amino acids; and (v) missense changes predicted to be damaging by at least one prediction program (CADD, PolyPhen-2, SIFT or PROVEAN). Based on familial consanguinity, homozygous variants were prioritized, leaving 9,769 changes of which 4,798 had putative functional consequence. After filtering for novelty or very low frequency (<1%; without any reported homozygosity) in publicly available databases and selecting only variants within the five identified homozygous regions, 10 changes remained (**Table S4**). Five gene variants (*PLOD2, HPS3, EIF2A, SMTNL2, POTEC*) were predicted to be benign, tolerated, or neutral by CADD^15^, PolyPhen-2, SIFT, PROVEAN and Mutation Taster (**Table S4**) and did not segregate with the disease phenotype within the family, so were therefore excluded from further analysis. The remaining five genes both segregated with the disease phenotype and were predicted deleterious by two or more predictors (**Table S4**). These comprised four missense mutations (*AGTR1*, *SLC6A7*, *EXOSC2*, *MED27)* and one nonsense mutation (*MPPE1*). *AGTR1* and *EXOSC2* were deemed to be unlikely candidates given their association with phenotypes not apparent in the patients (cardiovascular/renal disease and short stature/hearing loss/retinitis pigmentosa respectively) (**Table S4**). Of the remaining three genes, *MED27* appeared to be the most likely candidate: the variant affects a highly conserved residue (**Figure S1A**) and has been reported in patients with similar disease phenomenology^16^. However, given the brain-dominant expression pattern of the two other genes, and compelling nature of both variants (**Table S5**), *SLC6A7* and *MPPE1* could not be excluded as candidate genes. To ensure that there were no other relevant variants, we also interrogated the WES data for reported genes associated with neurological disease but no relevant pathogenic variants were identified (**Table S6,S7**).

**C: Supplementary Tables**

**Table S1: Exome and genome patient cohorts**

| **Cohort** | **Reference/website** |
| --- | --- |
| The UK10K project and IHR BioResource-Rare Diseases (NIHRBR-RD) study (1151 exomes/genomes) | ^17,18^ |
| Deciphering Developmental Disorders (DDD) study (4,295 triomes) through a complementary research proposal (CAP 97) | ^19^ |
| The NIH Undiagnosed Diseases Program, Bethseda, USA | ^18^ |
| Radboud University Medical Centre, Nijmegen, Netherlands |  |
| Institut für Humangenetik, München, Germany |  |
| Gene Matcher | https://genematcher.org/ |

**Table S3:** **Homozygous regions and potential disease loci shared by the two affected siblings**

| Chr | Start^a^ | End^a^ | first SNP | last SNP | Size [Mb] | Genes in region  (Candidate genes in bold) |
| --- | --- | --- | --- | --- | --- | --- |
| 3 | 144,639,871 | 153,477,976 | rs6766160 | rs9812437 | 8.84 | *PLOD2,* ***AGTR1****, HPS3, EIF2A* |
| 5 | 146,780,521 | 153,009,489 | rs13168262 | rs1422884 | 6.23 | ***SLC6A7*** |
| 9 | 132,995,160 | 137,761,104 | rs7852859 | rs4073505 | 4.77 | ***EXOSC2*** |
| 17 | 3,423,439 | 12,049,697 | rs17763099 | rs12938468 | 8.63 | ***MED27*** |
| 18 | 1,937,231 | 38,662,458 | rs4797949 | rs16974043 | 20.84 | ***MPPE1****, POTEC* |

^a^based on NCBI build 37.1; Chr: chromosome

**Table S4: Rare variants identified on whole exome sequencing located in homozygous regions of linkage**

| **Chr** | **Gene**  **(Transcript)** | **cDNA change** | **Protein change** | **Coding effect** | **dbSNP ID**  **(MAF)** | **GnomAD Hom count** | **CADD** | **PolyPhen2** | | **SIFT** | **PROVEAN** | **Mutation Taster** | **Segregation** | **Associated phenotype(s)** | **Disease gene candidate?**  **Y/N**  **Reasoning** |
| --- | --- | --- | --- | --- | --- | --- | --- | --- | --- | --- | --- | --- | --- | --- | --- |
|  |  |  |  |  |  |  |  | HumDiv | HumVar |  |  |  |  |  |  |
| **3** | *PLOD2*  (NM_000935.2) | c.1669A>G | p.Ile557Val | Missense | rs558336915  (C=0.0003) | 1 | 14.95 | B (0.000) | B (0.003) | T (0.84) | N  (-0.286) | DC  (0.811) | - | AR Bruck syndrome 2 (MIM_ 609220) | N  Does not segregate appropriately  *In silico* predictions benign  Reported phenotype not in keeping with patients |
| **3** | *AGTR1*  (NM_000685.4) | c.344G>C | p.Ser115Thr | Missense | rs550259107  (C=0.0002) | 0 | 23.1 | D (0.973) | P (0.850) | T (0.22) | N  (-2.425) | DC (0.999) | Yes | AR Renal tubular dysgenesis (RTD) (MIM_ 267430) | N  *In silico* prediction are ambiguous  Reported phenotype not in keeping with patients |
| **3** | *HPS3*  (NM_032383.3) | c.2899A>G | p.Ile967Val | Missense | rs200136665  (G=0.000016) | 0 | 1.392 | B (0.000) | B (0.001) | T  (1) | N  (0.142) | P  (0.999) | - | AR Hermansky-Pudlak syndrome 3 (MIM_ 614072) | N  Does not segregate appropriately  *In silico* predictions benign  Reported phenotype not in keeping with patients |
| **3** | *EIF2A*  (NM_032025.4) | c.1663G>A | p.Ala555Thr | Missense | rs773552578  (A=0.000048) | 0 | 24.9 | B (0.444) | B (0.266) | T  (0.02) | N  (-1.197) | DC  (0.999) | - | N/A | N  Does not segregate appropriately  *In silico* predictions benign  Low expression in brain/neurones |
| **5** | *SLC6A7*  (NM_014228.3) | c.1186G>A | p.Gly396Ser | Missense | rs781233389  (A=0.000032) | 0 | 30 | D (1.000) | D (1.000) | D  (0) | D  (-5.761) | DC  (0.999) | Yes | N/A | Y  Segregates appropriately  *In silico* predictions damaging by all tools  Brain-specific expression |
| **9** | *EXOSC2*  (NM_014285.6) | c.185G>C | p.Arg62Thr | Missense | rs748977410  (C=0.00004) | 0 | 26.6 | D (0.987) | P (0.907) | D (0.03) | D  (-3.411) | DC  (0.999) | Yes | AR Short stature, hearing loss, retinitis pigmentosa, and distinctive facies (MIM_ 617763) | N  Reported phenotype not in keeping with patients |
| **9** | *MED27*  (NM_004269.3) | c.839C>T | p.Pro280Leu | Missense | rs778593272  (A=0.000053) | 0 | 25 | P (0.727) | B (0.097) | D (0.01) | D  (-6.741) | DC  (0.999) | Yes | N/A | Y  Segregates appropriately  *In silico* prediction are largely in favour  Ubiquitous expression |
| **17** | *SMTNL2*  (NM_001114974.1) | c.641C>T | p.Ala214Val | Missense | rs778243560  (T=0.000028) | 0 | 15.02 | B  (0.226) | B  (0.017) | T  (0.16) | N  (-0.319) | P  (0.999) | - | N/A | N  Does not segregate appropriately  *In silico* predictions benign  Skeletal muscle-specific expression  Hypothesised skeletal muscle function  Affected amino acid weakly conserved |
| **18** | *MPPE1*  (NM_023075.5) | c.985A>T | p.Arg329* | Nonsense | rs766824046  (A=0.000004) | 0 | 40 | N/A | N/A | N/A | D  (-121.151) | D  (1) | Yes | N/A | Y  Segregates appropriately  *In silico* predictions damaging by all tools  Expressed in brain |
| **18** | *POTEC*  (NM_001137671.1) | c.1352G>A | p.Arg451Lys | Missense | N/A | N/A | 0.002 | B (0.000) | B (0.000) | T  (1) | N  (0.619) | P  (0.999) | - | N/A | N  Does not segregate appropriately  *In silico* predictions benign  Testes-specific expression |

Chr: chromosome; B: benign; D: probably damaging (PolyPhen2) or damaging (SIFT) or deleterious (PROVEAN); N: neutral; P: possibly damaging; T: tolerated.

**Table S5: Expression pattern and putative function of candidate genes**

| Gene | Full name | Function | Expression | Mouse models | Associated human phenotypes |
| --- | --- | --- | --- | --- | --- |
| *AGTR1* | Angiotensin II receptor, type 1 | Angiotensin II is potent vasopressor hormone and primary regulator of aldosterone secretion, important effector controlling blood pressure and volume in cardiovascular system; type 1 receptor thought to mediate major cardiovascular effects of angiotensin II | Liver, lung, adrenal and adrenocortical adenomas | Homozygotes for the Agtr1b/Agtr1a double knockout exhibit reductions in growth, survival, blood pressure, and kidney size not found in either single knockout | Hypertension (OMIM #145500)  Renal tubular dysgenesis  (OMIM #267430) |
| *SLC6A7* | Solute carrier family 6 (neurotransmitter transporter), member 7 | Na^+^/Cl^-^ dependent plasma membrane transporter of proline; presynaptic regulatory role in excitatory synaptic transmission; transporter for an excitatory neurotransmitter of L-proline | Brain | No phenotypes | Not reported |
| *EXOSC2* | Exosome component 2 | Required for 3-prime to 5-prime exonuclease activity generating 3-prime end of 5.8S rRNA; component of mRNA decay complex | Widely | No phenotypes | Short stature, hearing loss, retinitis pigmentosa (OMIM #617763) |
| *MED27* | Mediator complex subunit 27 | Global coactivator for nuclear receptor family; component of Mediator complex, coactivator involved in regulated transcription of RNA polymerase II-dependent genes | Widely | No phenotypes | Neurodevelopmental disorder, spasticity, cataracts, cerebellar hypoplasia (OMIM #619286) |
| *MPPE1* | Metallophosphoesterase 1 | Metallophosphoesterase required for transport of GPI-anchor proteins from endoplasmic reticulum to Golgi; acts in lipid remodeling steps of GPI-anchor maturation | Brain | No phenotypes | Not reported |

**Table S6: Genes causing neurodevelopmental syndromes, movement disorders, and epilepsy phenotypes, interrogated on whole-exome sequencing**
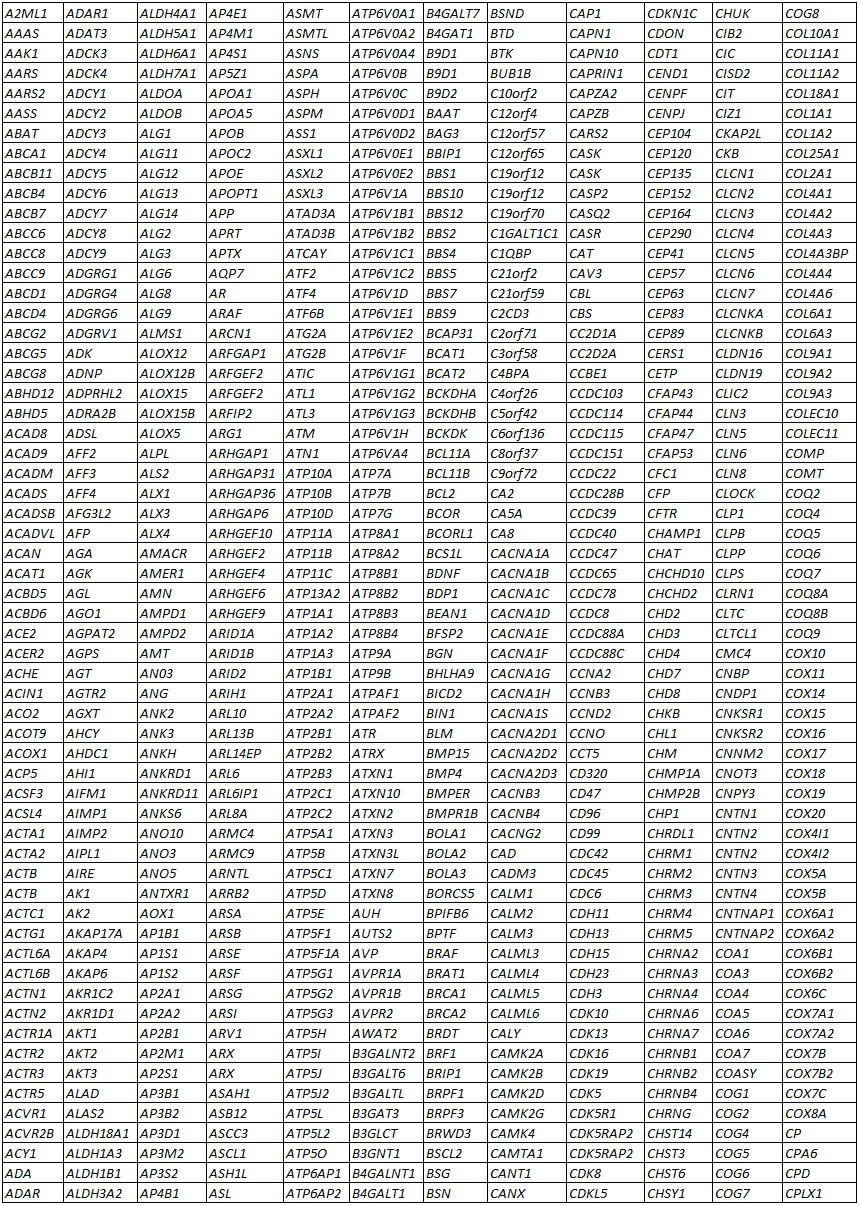

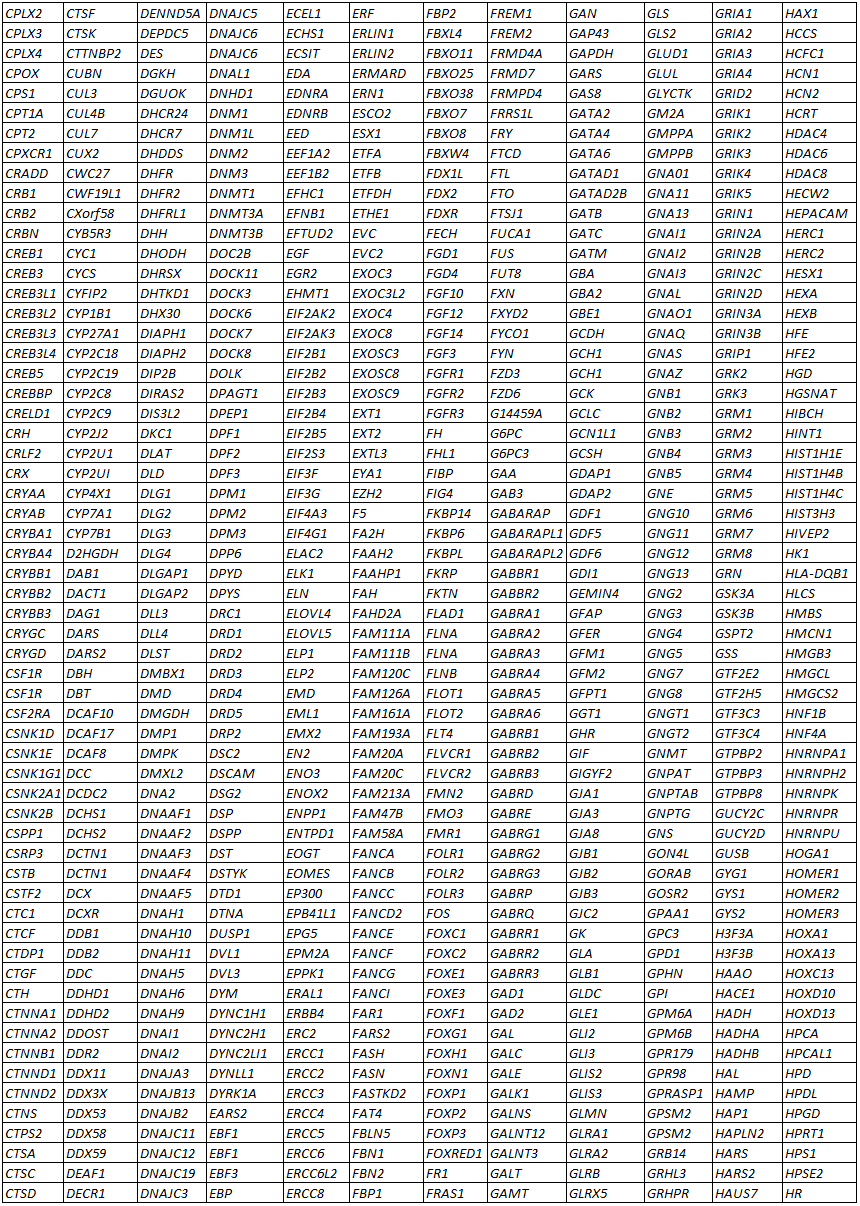

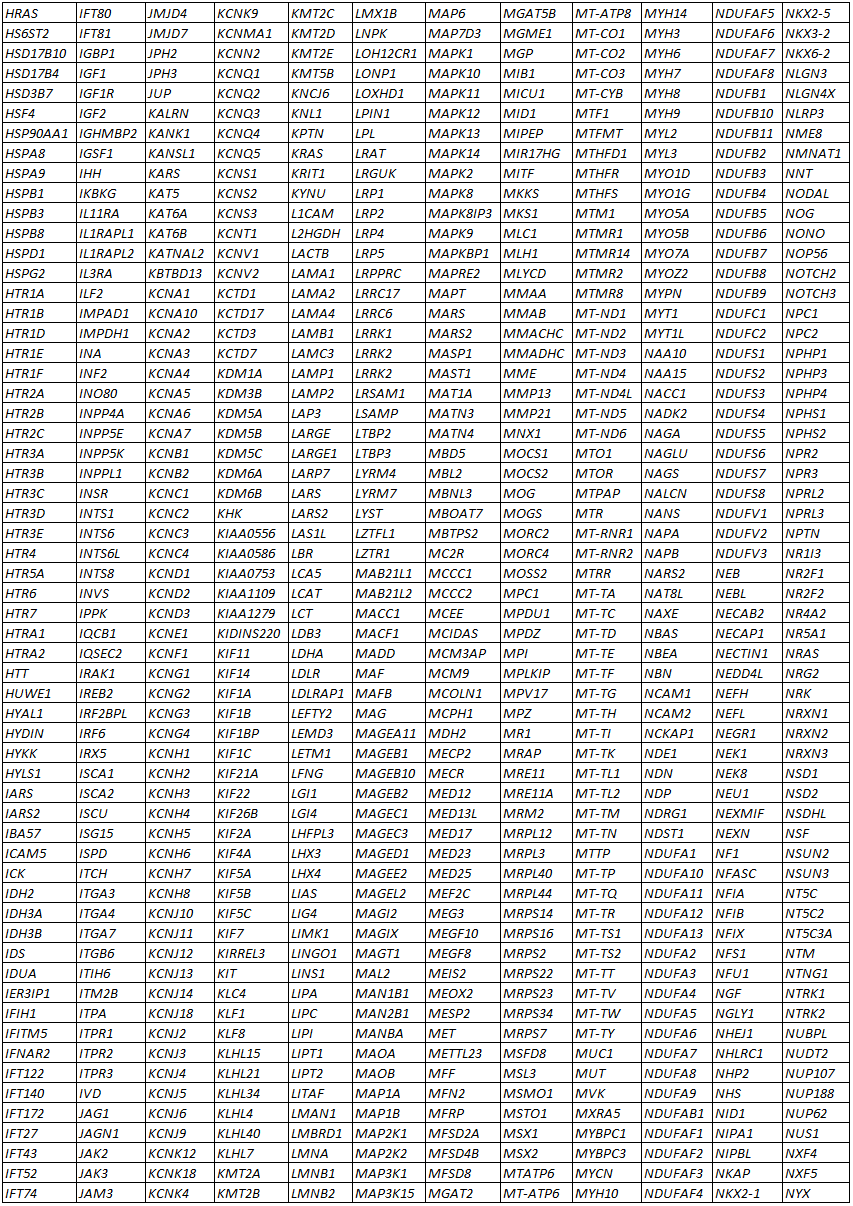

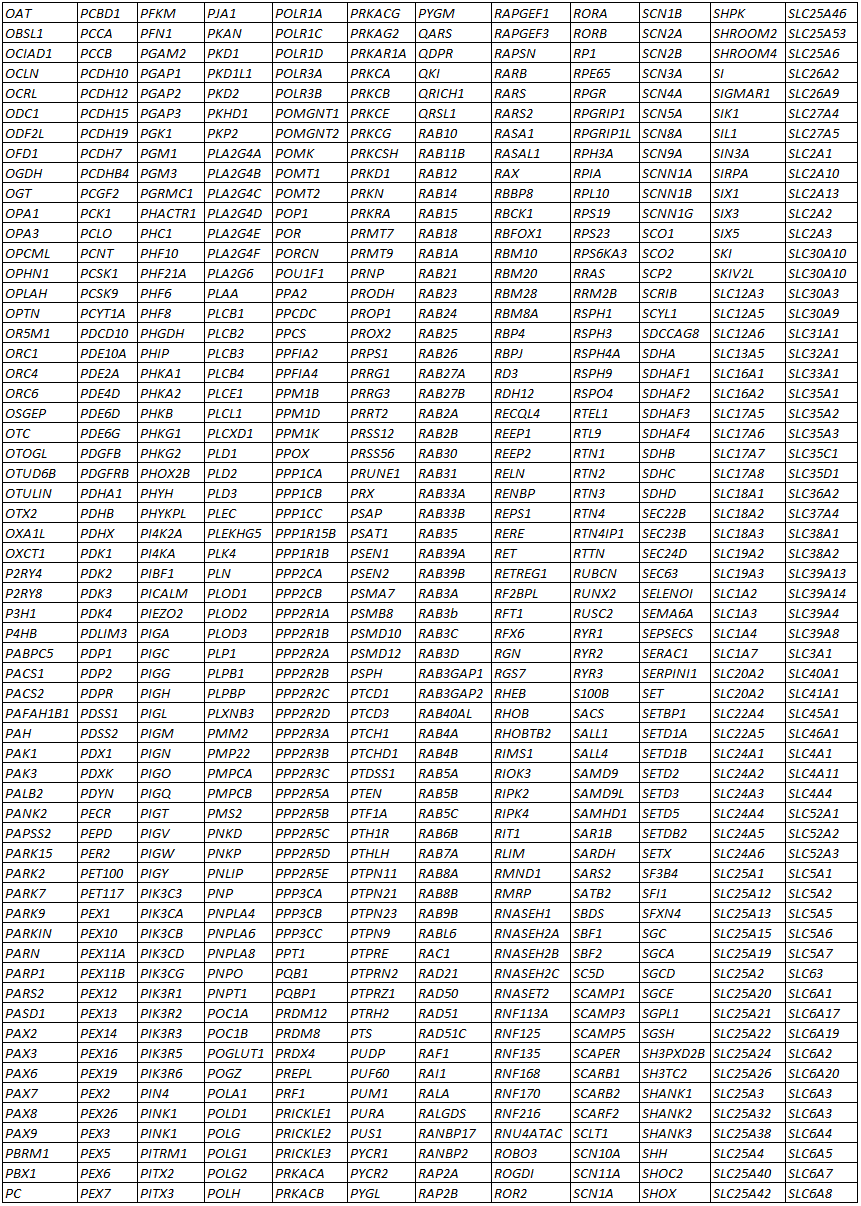

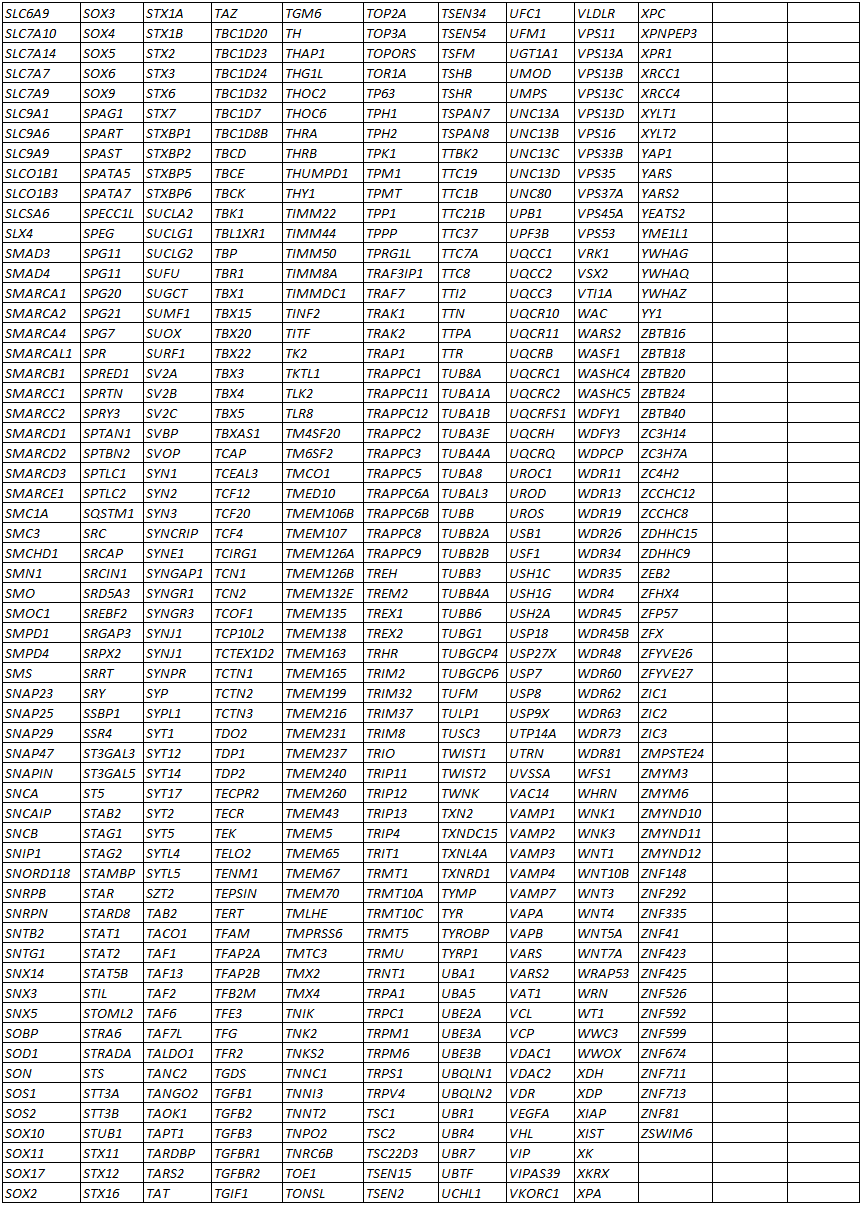


**Table S7: Reported cataract genes interrogated on whole-exome sequencing**


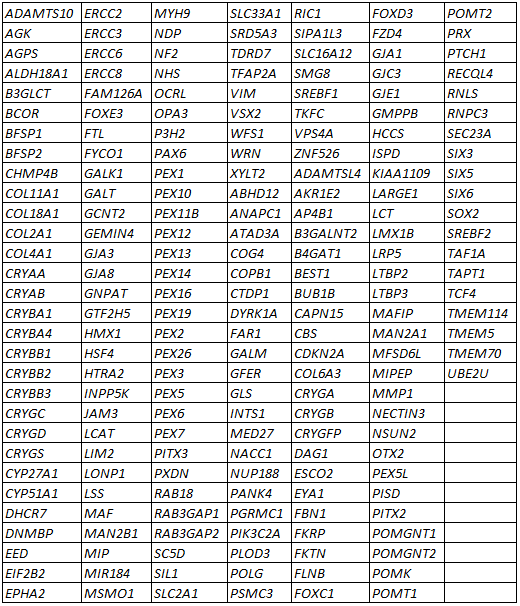


**Table S8:** Phenotypic information for the reported cases compared to previously reported *MED27* cases (differences highlighted)

| **Feature** | ***Meng et al. 2021***  **Summary of 11 families** | **This report** | |
| --- | --- | --- | --- |
|  |  | **II:1** | **II:2** |
| **Disease severity** | Severe (8)  Moderate (5) | Severe | Severe |
| **Gender** | Male (5)  Female (11) | Male | Female |
| **Consanguinity** | Yes, in 5/11 families | Yes | Yes |
| **Birth history** | Normal in 13/15 | Normal | Normal |
| **Microcephaly** | Yes – in 4/14 | Yes | Yes |
| **Feeding issues** | 6/14 had swallowing difficulties | GI dysmotility | GI dysmotility |
| **Motor delay** | Present in 13/15 | Yes  Unable to sit/walk | Yes  Unable to sit/walk |
| **Language delay** | Present in 13/15 | Yes  Non-verbal | Yes  Non-verbal |
| **Intellectual disability** | Yes, in all | Severe | Severe |
| **Developmental regression** | Present in 5/15 | Yes | Yes |
| **Central hypotonia** | Present in 14/15 | Yes | Yes |
| **Distal spasticity** | Present in 13/15 |  |  |
| **Dystonic movement** | Present in 13/15  No status dystonicus reported | Yes  Recurrent status dystonicus | Yes  Recurrent status dystonicus |
| **Chorea** | Not reported | Yes | Yes |
| **Epilepsy** | Present in 9/15 | Yes  Onset age 4 | Yes  Onset age 4 |
| **Brain MRI cerebellar atrophy** | Present in 12/14 | Yes | Yes |
| **Brain MRI others** | No striatal atrophy | Vermian aplasia. Symmetrical striatal atrophy. Bilateral frontal lobe atrophy | Vermian aplasia |
| **Cataracts** | Present in 10/15 | Yes  Early childhood, lensectomy | Yes  Early childhood, lensectomy |
| **Dysmorphic features** | None consistent across cohort | Distinctive facial features with prominent eyebrows, a slightly pointed nose, almond-shaped eyes, and unusually shaped, borderline low-set ears | |
| **MED27 (NM_004269.3)** | Homozygous or compound heterozygous variants (*)   c.188T>G (p. p.Val63Gly)*  c.298_302del (p. p.Lys100Serfs*4)*  c.392_393del (p. p.Gln131Leufs*7)*  c.565-566del (p. p.M189Afs*21)*  c.682-2A>G (p.?)*  c.695C>T (p.Ser232Phe)*  c.725T>C (p.Val242Ala)  c.776C>T (p.Pro259Leu)*  c.839C>T (p.Pro280Leu)*  c.871G>A (p.Gly291Ser)*  c.878C>T (p.Pro293Leu) | Homozygous c.839C>T (p.Pro280Leu) | Homozygous c.839C>T (p.Pro280Leu) |

**Table S9: Members of the SLC6 transporter family**

| Subfamily | Gene | Major substrates | Tissue expression^a^ | Associated phenotype^b^ | Ref |
| --- | --- | --- | --- | --- | --- |
| GABA transporters | *SLC6A1*; GAT1 | GABA | **Brain**, bladder, liver parathyroid | **Myoclonic-atonic epilepsy** (MIM #616421) | ^20^ |
|  | *SLC6A6*; TauT | Taurine, β-alanine | Ubiquitous | **Dilated Cardiomyopathy** (MIM #145350) | ^21–23^ |
|  | *SLC6A8*; CT1 | Creatine | Ubiquitous | **Cerebral creatine deficiency syndrome 1** (MIM #300352) with X-linked intellectual disability | ^24^ |
|  | *SLC6A11*; GAT3 | GABA | **Brain**, eye, spinal cord | None reported | ─ |
|  | *SLC6A12*; BGT1 | Betaine, GABA | **Kidney**, brain, liver | None reported | ─ |
|  | *SLC6A13*; GAT2 | GABA | **Kidney, liver**, brain eye | None reproted | ─ |
| Monoamine transporters | *SLC6A2*; NET | Noradrenaline, Dopamine | **Brain, adrenal gland**, intestine, kidney, placenta testis | **Orthostatic intolerance** (MIM #604715) | ^25^ |
|  | *SLC6A3*; DAT | Dopamine | **Brain**, thymus | **Parkinsonism-dystonia, infantile** (MIM #613135);  Association with autism spectrum disorder  Association with Attention-Deficit Hyperactivity Disorder (MIM#143465)  Association with adult parkinsonism  Association with Bipolar disorder (MIM #125480)  Association with Cigarette smoking (MIM #188890) | ^3^  ^26^  ^27^  ^28^  ^29^  ^29,30^ |
|  | *SLC6A4*; SERT | Serotonin | **Brain**, bone, intestine, thymus | Association with Anxiety-related personality traits (MIM #607834)  Association with Obsessive-compulsive disorder (MIM #164230) | ^31^  ^32^ |
| Amino acid transporters (I) | *SLC6A5*; GlyT2 | Glycine | **Spinal cord**, brain, eye | **Startle disease/Hyperekplexia 3** (MIM #614618) | ^33^ |
|  | *SLC6A7*; PROT | Proline | **Brain** | Association with early-onset neurodevelopmental disorder with hyperkinesia and epilepsy | This study |
|  | *SLC6A9*; GlyT1 | Glycine | Widely | **Glycine encephalopathy with normal serum glycine** (MIM #617301) | ^34,35^ |
|  | *SLC6A14*; ATB^0,+^ | Neutral, cationic amino acids | **Lung**, pituitary, colon, mammary gland | Association with Obesity (MIM #300306) | ^36^ |
| Amino acid transporters (II) | *SLC6A15*; B^0^AT2 | BCAA, Met, Pro | **Brain**, eye, muscle placenta | Association with Major depression disorder 1 (MIM #608520) | ^37^ |
|  | *SLC6A16*; NTT5 | Unknown | **Testis**, blood, bone | None reported | ─ |
|  | *SLC6A17*; NTT4 | BCAA, Met, Pro, Ala, Gln | **Brain**, eye, pituitary, pancreas | **Intellectual disability with progressive tremor, speech impairment, and behavioral problems** (MIM #616269) | ^38^ |
|  | *SLC6A18*; B^0^AT3 | Gly, Ala | **Kidney** | None reported | ─ |
|  | *SLC6A19*; B^0^AT1 | Neutral amino acids | **Kidney, intestine**, skin | **Hartnup disorder** (MIM #234500)  Association with Hyperglycinuria (MIM #138500)  Association with Iminoglycinuria, digenic (MIM #242600) | ^39,40^  ^41^  ^41^ |
|  | *SLC6A20*; SIT1 | Proline, betaine | **Intestine**, brain, eye | Association with Hyperglycinuria (MIM #138500)  Association with Iminoglycinuria, digenic (MIM #242600) | ^41^  ^41^ |

^a^in bold indicates main organ of expression; ^b^in bold are monogenic disorders; BCAA: branched chain amino acid; Ref: reference

**Table S10: Primers used for confirmation of variants identified on whole exome sequencing**

| Primer name | Sequence (5’ → 3’) | Amplicon size (bp) | Annealing temp (°C) |
| --- | --- | --- | --- |
| AGTR1_3.2F  AGTR1_3.2R | tggccagtgtttttcttttg  ccttctttagggccttccaa | 489 | 60 |
| SLC6A7_9F  SLC6A7_9R | taagtggccgtgtgtgtctg  ggtcagaattctatccccaaca | 299 | 60 |
| EXOSC2_2F  EXOSC2_2R | gggagcctgttagctttgag  ggggtgggggataaggag | 287 | 60 |
| MED27_c839_F  MED27_c839_R | aatcacagggcatcttctgg  aacacaagggctcattccag | 300 | 60 |
| MPPE1_10F  MPPE1_10R | gagagcctggttgaggatga  gagtgtaagaggagaggatacgg | 297 | 55 |

**Table S11: qPCR primers**

| Primer name | Sequence (5’ → 3’) |
| --- | --- |
| MPPE1 F  MPPE1 R | tcacaaaagctgctgtgg  tctgtgggcgtgatgcta |
| GAPDH F  GAPDH R | ctctgctcctcctgttcgac acgaccaaatccgttgactc |

**D. Suplementary Figure Legends**

**Figure S1: *MED27*, *SCL6A7* and *MPPE1* variant conservation**

Sequence alignment of amino acids of indicated proteins in Human, Chimp, Rat, Mouse, Platypus, Chicken, Zebrafish and Fruit fly. Black boxes indicate alternate amino acid in each gene variant. (**A**) Alignment of MED27 amino acid sequences (**B**) PROT (*SLC6A7*) amino acid sequences. (**C**) Alignment of PGAP5 (*MPPE1*) amino acid sequence. Sequence after the variant will not be translated.

**Figure S2: Gene and protein schematic of *MPPE1*/PGAP5**

Top: Gene schematic of *MPPE1,* showing c.985A>T variant, 24bp before the end of the penultimate exon. Bottom: Schematic represents location of p.Arg329* variant upstream of key transmembrane domain and KxKxx ER retrieval motif.

**Figure S3: Biotinylation assays and Western blots of p.Gly396Ser variant overexpressed in HEK293T cells**

Three bands were observed; a band at 60 kDa corresponding to non-glycosylated PROT, and bands at 68kDa and 100kDa corresponding to glycosylated and/or phosphorylated PROT^42^. β-actin and Na^+^/K^+^ 𝛼 ATPase subunit were used as loading controls for total and cell surface expression respectively (P<0.05, Student’s t-test).

**Figure S4: Developmental stage of *slc6a7* morphants**

*slc6a7* morphants expressing human expressing either PROT-WT or PROT-G396S at 22 and 34 hpf were characterized in terms of their developmental stage. PROT-WT RNA rescued development delay seen in the *slc6a7* morphants.

**Figure S5: Schematic of PGAP5 function.**

PGAP5 is present in the Golgi, where it translocates to the ER membrane in order to remove an ethanolamine-phosphate group from the second mannose group on the GPI anchor. The GPI-AP is then able to move from the ER to the Golgi, where it can exit and translocate to the cell membrane.

**Figure S6: Flow cytometry plots of cell-surface GPI-AP expression (CD73, CD109 and FLAER) in patient fibroblasts.**

Unstained cells are shown in blue; negative control cells in orange, green and red; patient cells in purple and brown; positive control cells in pink.

**E: Supplementary References**

1. Quinlan AR, Hall IM. BEDTools: A flexible suite of utilities for comparing genomic features. *Bioinformatics*. 2010;26(6):841-842. doi:10.1093/bioinformatics/btq033

2. Untergasser A, Cutcutache I, Koressaar T, et al. Primer3-new capabilities and interfaces. *Nucleic Acids Res*. 2012;40(15):1-12. doi:10.1093/nar/gks596

3. Kurian MA, Zhen J, Cheng SY, et al. Homozygous loss-of-function mutations in the gene encoding the dopamine transporter are associated with infantile parkinsonism-dystonia. *J Clin Invest*. 2009;119(6):1595-1603. doi:10.1172/JCI39060

4. Kurian MA, Li Y, Zhen J, et al. Clinical and molecular characterisation of hereditary dopamine transporter deficiency syndrome: An observational cohort and experimental study. *Lancet Neurol*. 2011;10(1):54-62. doi:10.1016/S1474-4422(10)70269-6

5. Livak KJ, Schmittgen TD. Analysis of relative gene expression data using real-time quantitative PCR and the 2-ΔΔCT method. *Methods*. 2001;25(4):402-408. doi:10.1006/meth.2001.1262

6. Varadi M, Anyango S, Deshpande M, et al. AlphaFold Protein Structure Database: massively expanding the structural coverage of protein-sequence space with high-accuracy models. *Nucleic Acids Res*. 2022;50(D1):D439-D444. doi:10.1093/nar/gkab1061

7. Jumper J, Evans R, Pritzel A, et al. Highly accurate protein structure prediction with AlphaFold. *Nature*. 2021;596(7873):583-589. doi:10.1038/s41586-021-03819-2

8. Pettersen EF, Goddard TD, Huang CC, et al. UCSF Chimera - A visualization system for exploratory research and analysis. *J Comput Chem*. 2004;25(13):1605-1612. doi:10.1002/jcc.20084

9. Shapovalov M V., Dunbrack RL. A smoothed backbone-dependent rotamer library for proteins derived from adaptive kernel density estimates and regressions. *Structure*. 2011;19(6):844-858. doi:10.1016/j.str.2011.03.019

10. Lomize MA, Pogozheva ID, Joo H, Mosberg HI, Lomize AL. OPM database and PPM web server: Resources for positioning of proteins in membranes. *Nucleic Acids Res*. 2012;40(D1):370-376. doi:10.1093/nar/gkr703

11. Kimmel CB, Ballard WW, Kimmel SR, Ullmann B, Schilling TF. Stages of embryonic development of the zebrafish. *Dev Dyn*. 1995;203(3):253-310. doi:10.1002/aja.1002030302

12. Nguyen TTM, Murakami Y, Mobilio S, et al. Bi-allelic Variants in the GPI Transamidase Subunit PIGK Cause a Neurodevelopmental Syndrome with Hypotonia, Cerebellar Atrophy, and Epilepsy. *Am J Hum Genet*. 2020;106(4):484-495. doi:10.1016/j.ajhg.2020.03.001

13. Nguyen TTM, Murakami Y, Sheridan E, et al. Mutations in GPAA1, Encoding a GPI Transamidase Complex Protein, Cause Developmental Delay, Epilepsy, Cerebellar Atrophy, and Osteopenia. *Am J Hum Genet*. 2017;101(5):856-865. doi:10.1016/j.ajhg.2017.09.020

14. Mostyn SN, Wilson KA, Schumann-Gillett A, et al. Identification of an allosteric binding site on the human glycine transporter, GlyT2, for bioactive lipid analgesics. *Elife*. 2019;8:1-22. doi:10.7554/eLife.47150

15. Rentzsch P, Witten D, Cooper GM, Shendure J, Kircher M. CADD: Predicting the deleteriousness of variants throughout the human genome. *Nucleic Acids Res*. 2019;47(1):886-D894. doi:10.1093/nar/gky1016

16. Meng L, Isohanni P, Shao Y, et al. MED27 Variants Cause Developmental Delay, Dystonia, and Cerebellar Hypoplasia. *Ann Neurol*. 2021;89(4):828-833. doi:10.1002/ana.26019

17. The UK10K Consortium. The UK10K project identifies rare variants in health and disease. *Nature*. 2015;526:82-90. doi:10.1038/nature14962

18. Gahl WA, Markello TC, Toro C, et al. The NIH undiagnosed diseases program: Insights into rare diseases. *Genet Med*. 2012;14(1):51-59. doi:10.1038/gim.0b013e318232a005.The

19. The Deciphering Developmental Disorders Study, Fitzgerald TW, Gerety SS, et al. Large-scale discovery of novel genetic causes of developmental disorders. *Nature*. 2015;519(7542):223-228. doi:10.1038/nature14135.Large-scale

20. Carvill GL, McMahon JM, Schneider A, et al. Mutations in the GABA transporter SLC6A1 cause epilepsy with myoclonic-atonic seizures. *Am J Hum Genet*. 2015;96(5):808-815. doi:10.1016/j.ajhg.2015.02.016

21. Garnier S, Harakalova M, Weiss S, et al. Genome-wide association analysis in dilated cardiomyopathy reveals two new players in systolic heart failure on chromosomes 3p25.1 and 22q11.23. *Eur Heart J*. 2021;42(20):2000-2011. doi:10.1093/eurheartj/ehab030

22. Shakeel M, Irfan M, Khan IA. Rare genetic mutations in Pakistani patients with dilated cardiomyopathy. *Gene*. 2018;673:134-139. doi:10.1016/j.gene.2018.06.019

23. Ansar M, Ranza E, Shetty M, et al. Taurine treatment of retinal degeneration and cardiomyopathy in a consanguineous family with SLC6A6 taurine transporter deficiency. *Hum Mol Genet*. 2020;29(4):618-623. doi:10.1093/hmg/ddz303

24. Salomons GS, Van Dooren SJM, Verhoeven NM, et al. X-linked creatine-transporter gene (SLC6A8) defect: A new creatine-deficiency syndrome. *Am J Hum Genet*. 2001;68(6):1497-1500. doi:10.1086/320595

25. Shannon JR, Flattem NL, Jordan J, et al. Orthostatic intolerance and tachycardia associated with norepinephrine- transporter deficiency. *N Engl J Med*. 2000;342(8):541-549. doi:10.1056/NEJM200002243420803

26. Hamilton PJ, Campbell NG, Sharma S, et al. De novo mutation in the dopamine transporter gene associates dopamine dysfunction with autism spectrum disorder. *Mol Psychiatry*. 2013;18(12):1315-1323. doi:10.1038/mp.2013.102

27. Hansen FH, Skjørringe T, Yasmeen S, et al. Missense dopamine transporter mutations associate with adult parkinsonism and ADHD. *J Clin Invest*. 2014;124(7):3107-3120. doi:10.1172/JCI73778

28. Greenwood T, Alexander M, Keck P, et al. Evidence for linkage disequilibrium between the dopamine transporter and bipolar disorder. *Am J Hum Genet*. 2001;105(2):145-151. doi:10.1002/1096-8628(2001)9999:9999

29. Lerman C, Caporaso NE, Audrain J, et al. Evidence suggesting the role of specific genetic factors in cigarette smoking. *Heal Psychol*. 1999;18(1):14-20. doi:10.1037//0278-6133.18.1.14

30. Sabol S, Nelson ML, Fisher C, et al. A genetic association for cigarette smoking behavior. *Heal Psychol*. 1999;18(1):7-13. doi:https://doi.org/10.1037/0278-6133.18.1.7

31. Lesch K, Bengel D, Heils A, et al. Association of anxiety-related traits with a polymorphism in the serotonin transporter gene regulatory region. *Science (80- )*. 1996;274(5292):1527-1531. doi:10.1126/science.274.5292.1527

32. Ozaki N, Goldman D, Kaye WH, et al. Serotonin transporter missense mutation associated with a complex neuropsychiatric phenotype. *Mol Psychiatry*. 2003;8(11):933-936. doi:10.1038/sj.mp.4001365

33. Rees MI, Harvey K, Pearce BR, et al. Mutations in the gene encoding GlyT2 (SLC6A5) define a presynaptic component of human startle disease. *Nat Genet*. 2006;38(7):801-806. doi:10.1038/ng1814

34. Kurolap A, Armbruster A, Hershkovitz T, et al. Loss of Glycine Transporter 1 Causes a Subtype of Glycine Encephalopathy with Arthrogryposis and Mildly Elevated Cerebrospinal Fluid Glycine. *Am J Hum Genet*. 2016;99(5):1172-1180. doi:10.1016/j.ajhg.2016.09.004

35. Alfadhel M, Nashabat M, Qahtani H Al, et al. Mutation in SLC6A9 encoding a glycine transporter causes a novel form of non-ketotic hyperglycinemia in humans. *Hum Genet*. 2016;135(11):1263-1268. doi:10.1007/s00439-016-1719-x

36. Suviolahti E, Oksanen LJ, Öhman M, et al. The SLC6A14 gene shows evidence of association with obesity. *J Clin Invest*. 2003;112(11):1762-1772. doi:10.1172/JCI200317491

37. Kohli MA, Lucae S, Saemann PG, et al. The Neuronal Transporter Gene SLC6A15 Confers Risk to Major Depression. *Neuron*. 2011;70:252-265. doi:10.1016/j.neuron.2011.04.005

38. Iqbal Z, Willemsen MH, Papon MA, et al. Homozygous SLC6A17 mutations cause autosomal-recessive intellectual disability with progressive tremor, speech impairment, and behavioral problems. *Am J Hum Genet*. 2015;96(3):386-396. doi:10.1016/j.ajhg.2015.01.010

39. Kleta R, Romeo E, Ristic Z, et al. Mutations in SLC6A19, encoding B0AT1, cause Hartnup disorder. *Nat Genet*. 2004;36(9):999-1002. doi:10.1038/ng1405

40. Seow HF, Bröer S, Bröer A, et al. Hartnup disorder is caused by mutations in the gene encoding the neutral amino acid transporter SLC6A19. *Nat Genet*. 2004;36(9):1003-1007. doi:10.1038/ng1406

41. Bröer S, Bailey CG, Kowalczuk S, et al. Iminoglycinuria and hyperglycinuria are discrete human phenotypes resulting from complex mutations in proline and glycine transporters. *J Clin Invest*. 2008;118(12):3881-3892. doi:10.1172/JCI36625

42. Li L Bin, Chen N, Ramamoorthy S, et al. The role of N-glycosylation in function and surface trafficking of the human dopamine transporter. *J Biol Chem*. 2004;279(20):21012-21020. doi:10.1074/jbc.M311972200
