# Supplementary figures and images for: MED27, SLC6A7, and MPPE1 Variants in a Complex Neurodevelopmental Disorder with Severe Dystonia
Source: Mov Disord. 2022 Jul 25;37(10):2139–46. doi: 10.1002/mds.29147 (PMC9796674; doi:10.1002/mds.29147)

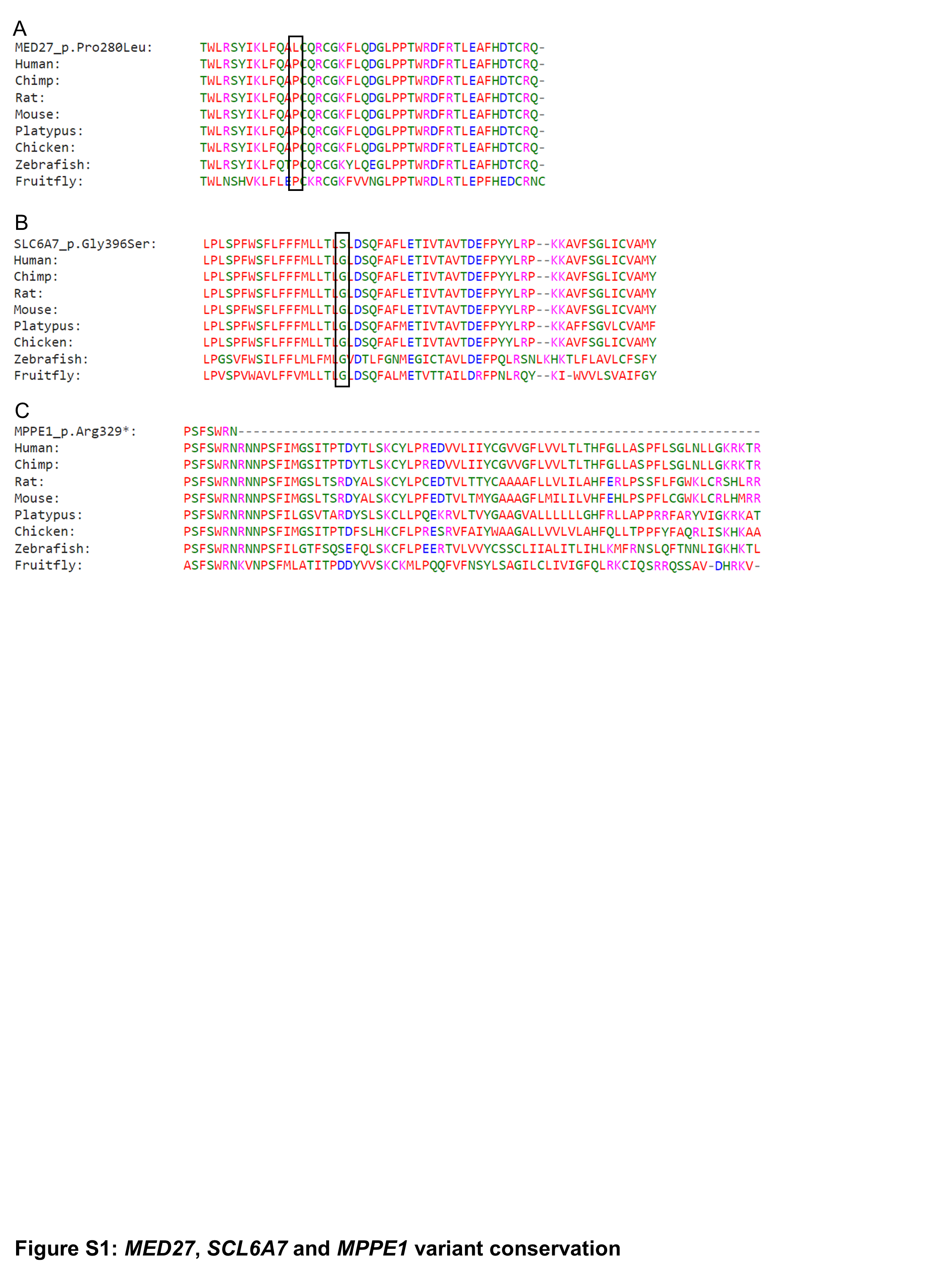

Supplement: Supplementary file 4 — FIG. S1 MED27, SCL6A7 and MPPE1 variant conservation. [file MDS-37-2139-s006.TIF]

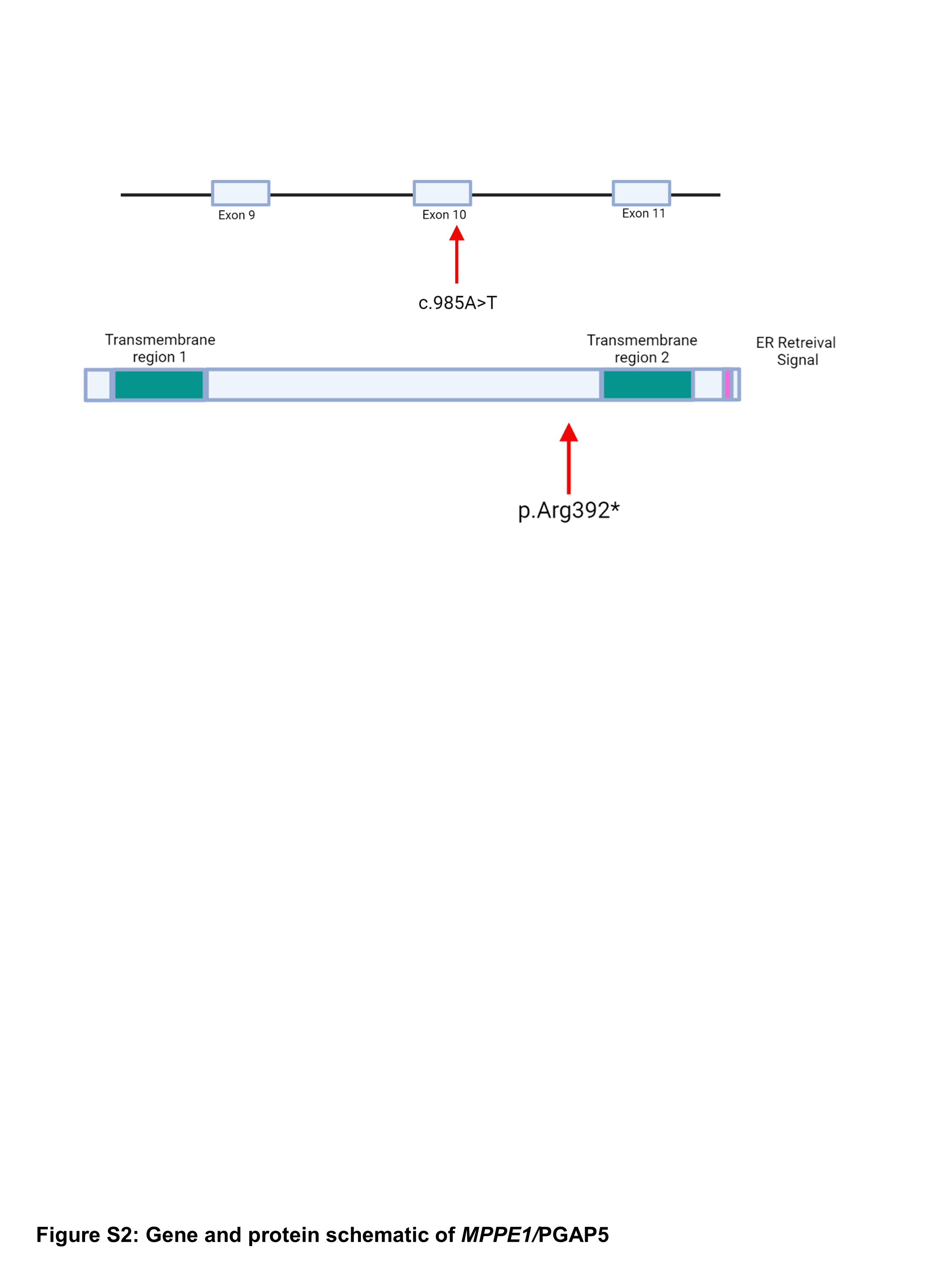

Supplement: Supplementary file 5 — FIG. S2 Gene and protein schematic of MPPE1/PGAP5. [file MDS-37-2139-s001.TIF]

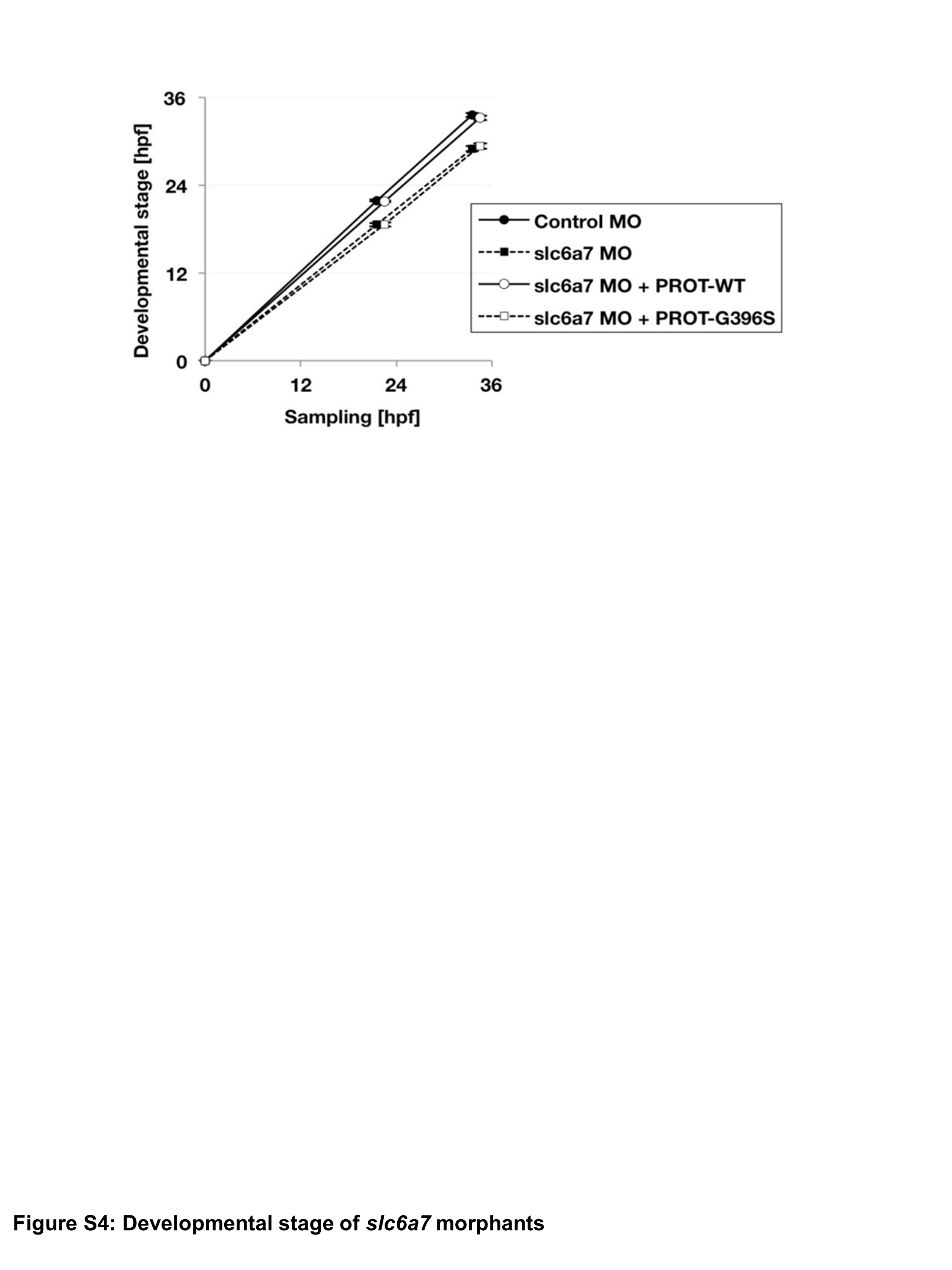

Supplement: Supplementary file 6 — FIG. S3 Biotinylation assays and Western blots of p.Gly396Ser variant overexpressed in HEK293T cells. [file MDS-37-2139-s009.TIF]

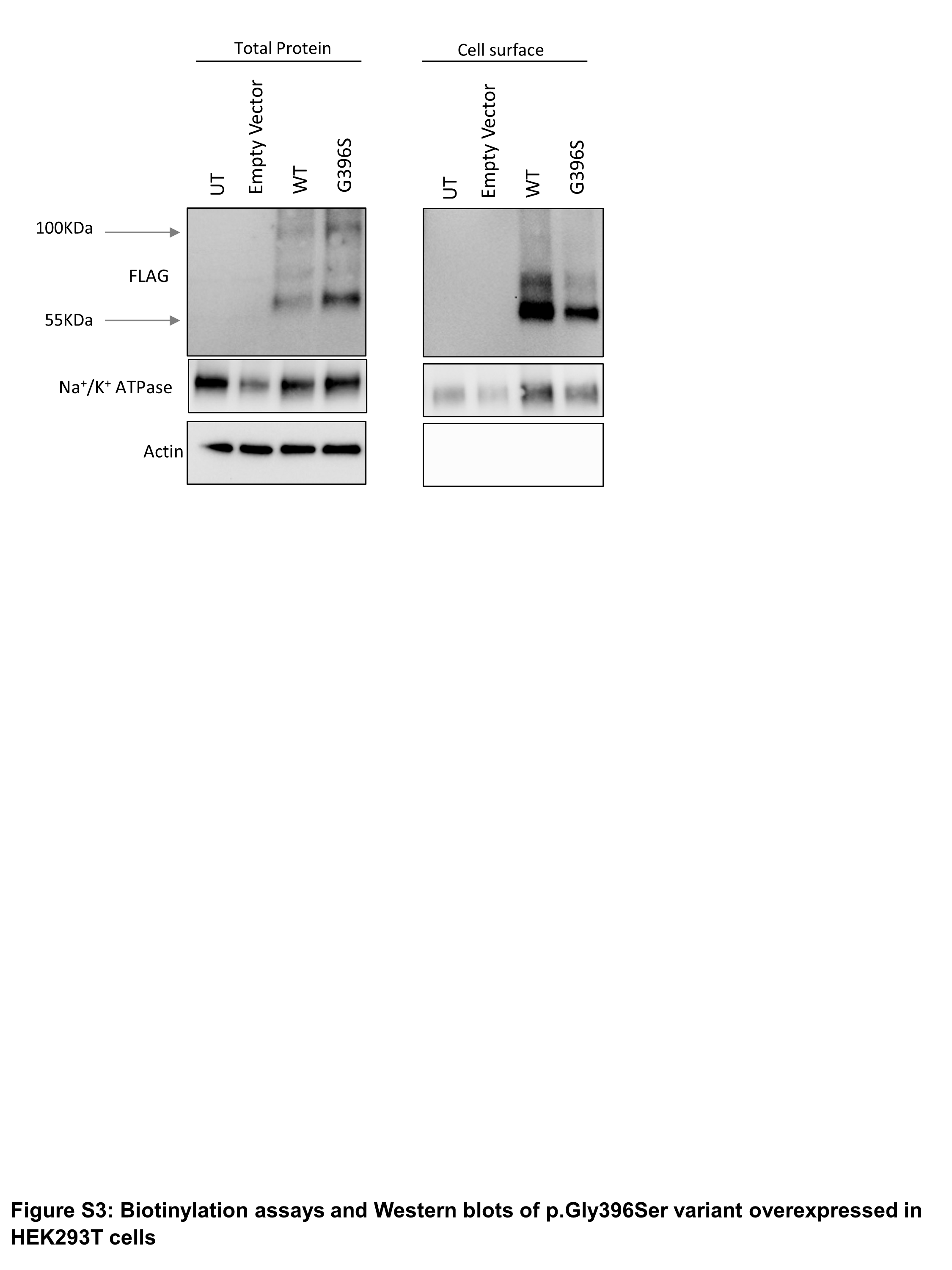

Supplement: Supplementary file 7 — FIG. S4 Developmental stage of sclc6a7 morphants. [file MDS-37-2139-s002.TIF]

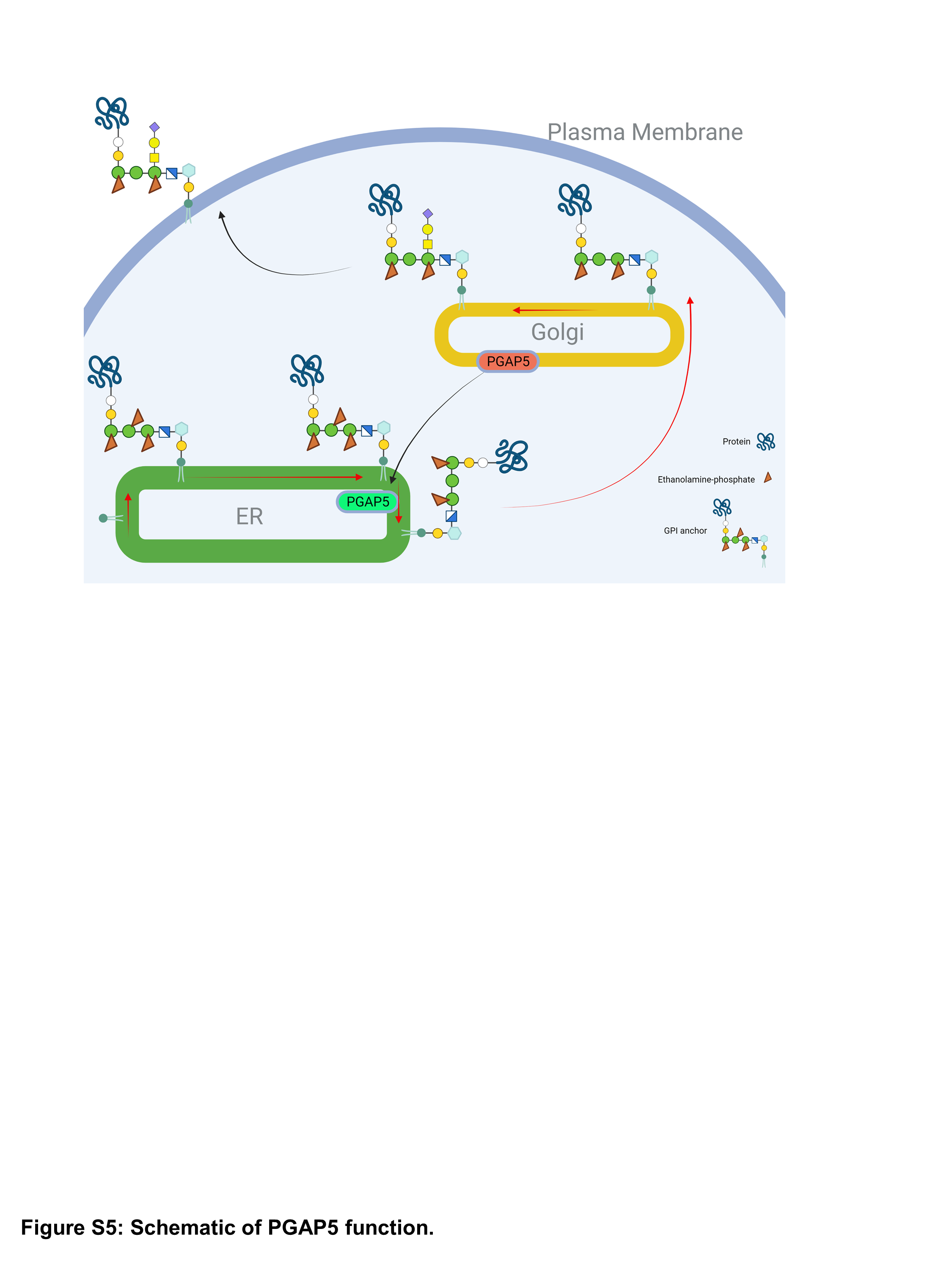

Supplement: Supplementary file 8 — FIG. S5 Schematic of PGAP5 function. [file MDS-37-2139-s005.TIF]

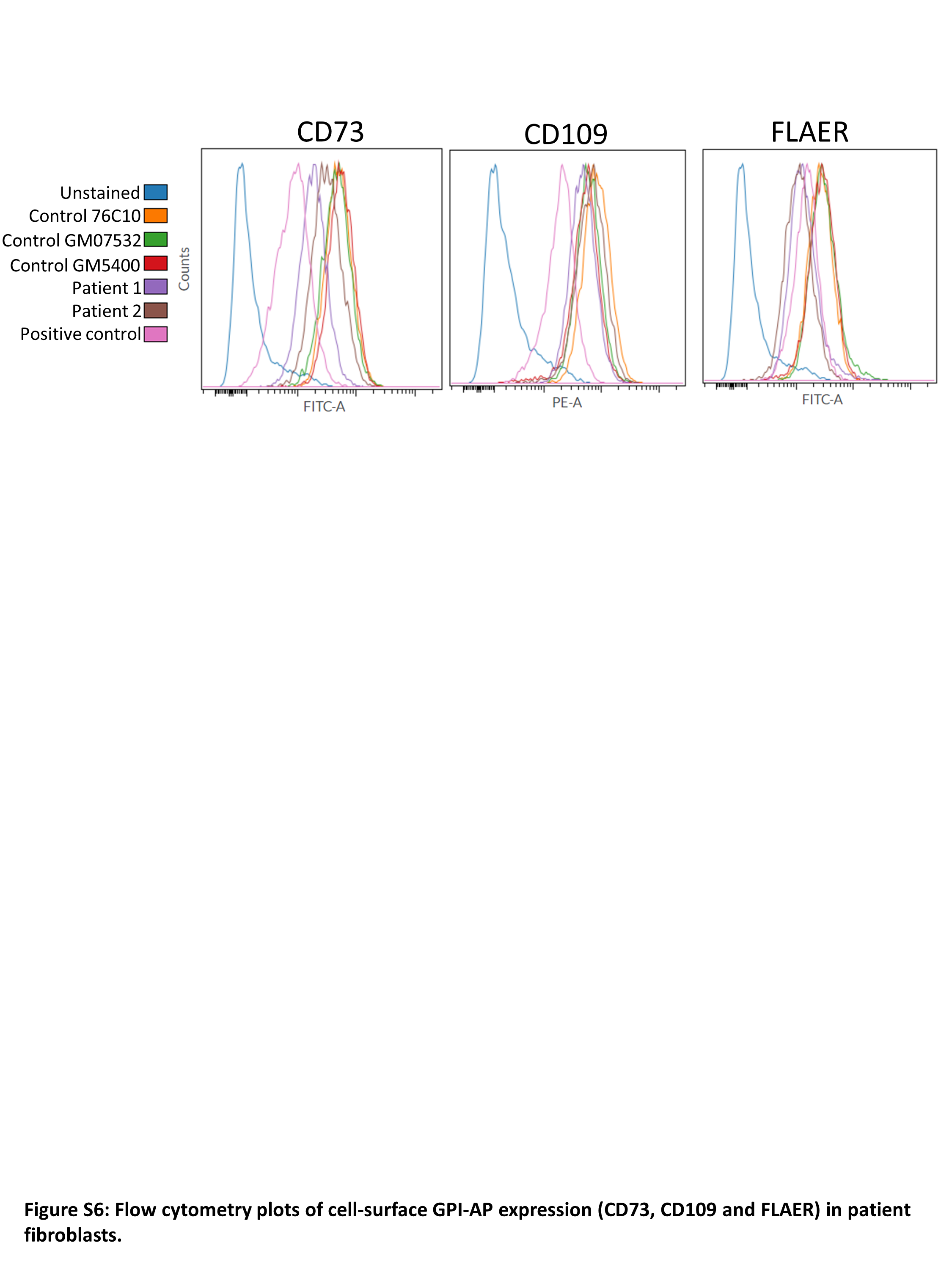

Supplement: Supplementary file 9 — FIG. S6 Flow cytometry plots of cell‐surface GPI‐AP expression (CD73, CD109 and FLAER) in patient fibroblasts. [file MDS-37-2139-s003.TIF]
